# A miR-192-EGR1-HOXB9 regulatory network controls the angiogenic switch in cancer

**DOI:** 10.1038/ncomms11169

**Published:** 2016-04-04

**Authors:** Sherry Y. Wu, Rajesha Rupaimoole, Fangrong Shen, Sunila Pradeep, Chad V. Pecot, Cristina Ivan, Archana S. Nagaraja, Kshipra M. Gharpure, Elizabeth Pham, Hiroto Hatakeyama, Michael H. McGuire, Monika Haemmerle, Viviana Vidal-Anaya, Courtney Olsen, Cristian Rodriguez-Aguayo, Justyna Filant, Ehsan A. Ehsanipour, Shelley M. Herbrich, Sourindra N. Maiti, Li Huang, Ji Hoon Kim, Xinna Zhang, Hee-Dong Han, Guillermo N. Armaiz-Pena, Elena G. Seviour, Sue Tucker, Min Zhang, Da Yang, Laurence J. N. Cooper, Rouba Ali-Fehmi, Menashe Bar-Eli, Ju-Seog Lee, Prahlad T. Ram, Keith A. Baggerly, Gabriel Lopez-Berestein, Mien-Chie Hung, Anil K. Sood

**Affiliations:** 1 Department of Gynecologic Oncology, The University of Texas MD Anderson Cancer Center, Houston, Texas 77030, USA; 2 Department of Obstetrics and Gynecology, The First Affiliated Hospital of Soochow University, Suzhou, Jiangsu Province 215006, China; 3 Division of Cancer Medicine, The University of Texas MD Anderson Cancer Center, Houston, Texas 77030, USA; 4 Department of Medicine, The University of North Carolina, Chapel Hill, North Carolina 27599 USA; 5 Center for RNA Interference and Non-Coding RNA, The University of Texas MD Anderson Cancer Center, Houston, Texas 77030, USA; 6 Biological Sciences Platform, Sunnybrook Research Institute, Toronto, Ontario, Canada, M4N 3M5; 7 Department of Experimental Therapeutics, The University of Texas MD Anderson Cancer Center, Houston, Texas 77030, USA; 8 Department of Cancer Biology, The University of Texas MD Anderson Cancer Center, Houston, Texas 77030, USA; 9 Department of Bioinformatics, The University of Texas MD Anderson Cancer Center, Houston, Texas 77030, USA; 10 Division of Pediatrics, The University of Texas MD Anderson Cancer Center, Houston, Texas 77030, USA; 11 Department of Systems Biology, The University of Texas MD Anderson Cancer Center, Houston, Texas 77030, USA; 12 Department of Immunology Laboratory, School of Medicine, Konkuk University, Chungju 380-701, South Korea; 13 Department of Pharmaceutical Sciences, University of Pittsburgh, Pittsburgh, Pennsylvania 15213, USA; 14 Department of Pathology, Wayne State University School of Medicine, Karmanos Cancer Institute, Detroit, Michigan 48201, USA; 15 Department of Molecular and Cellular Oncology, The University of Texas MD Anderson Cancer Center, Houston, Texas 77030, USA; 16 Center for Molecular Medicine, China Medical University, Taichung 40402, Taiwan

## Abstract

A deeper mechanistic understanding of tumour angiogenesis regulation is needed to improve current anti-angiogenic therapies. Here we present evidence from systems-based miRNA analyses of large-scale patient data sets along with *in vitro* and *in vivo* experiments that miR-192 is a key regulator of angiogenesis. The potent anti-angiogenic effect of miR-192 stems from its ability to globally downregulate angiogenic pathways in cancer cells through regulation of *EGR1* and *HOXB9*. Low miR-192 expression in human tumours is predictive of poor clinical outcome in several cancer types. Using 1,2-dioleoyl-sn-glycero-3-phosphatidylcholine (DOPC) nanoliposomes, we show that miR-192 delivery leads to inhibition of tumour angiogenesis in multiple ovarian and renal tumour models, resulting in tumour regression and growth inhibition. This anti-angiogenic and anti-tumour effect is more robust than that observed with an anti-VEGF antibody. Collectively, these data identify miR-192 as a central node in tumour angiogenesis and support the use of miR-192 in an anti-angiogenesis therapy.

## Abstract (web summary)

The formation of blood vessels in tumours, angiogenesis, is a promising target for therapy. Here, the authors show that microRNA192 has anti-angiogenic functions and negatively regulates *EGR1* and *HOXB9*, and that delivery of this microRNA to tumours *in vivo* can reduce angiogenesis and tumour growth.

## Graphical abstract

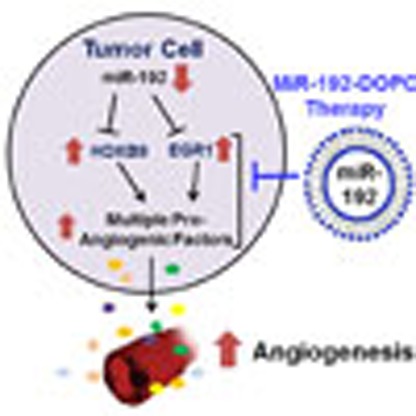

Angiogenesis plays a central role in tumour progression. However, targeting tumour vasculature has thus far resulted in only modest improvement in progression-free survival, with most patients still experiencing disease progression despite an initial response^1^,^2^,^3^. Emerging pre-clinical and clinical evidence indicates that upregulation of compensatory pro-angiogenic signalling cascades contributes significantly to the suboptimal therapeutic response in multiple cancer types, including ovarian and renal cancer^4^,^5^,^6^,^7^,^8^. In patients with glioblastoma, treatment with cediranib, a pan-VEGFR inhibitor, also resulted in subsequent increases in *FGF-2* and *SDF-1* levels, which can in turn contribute to tumour progression^1^,^6^. In a phase II trial of FOLFIRI+B (folinic acid, fluorouracil, irinotecan and bevacizumab) in colorectal cancer, significant increases in several pro-angiogenic factors were also observed following the treatment^5^. The identification of new anti-angiogenic approaches that can overcome this compensatory mechanism are, therefore, critical for improved therapeutic outcomes.

MicroRNAs (miRNAs) are endogenous small RNA molecules that have the ability to target multiple genes concurrently^9^. This unique property enables us to simultaneously target multiple important pathways involved in angiogenesis. Pathway redundancy is, thus, less likely to occur with such molecules. Despite the promise, a systematic evaluation of miRNAs that have a global impact on angiogenesis has not yet been reported. To address this knowledge gap, we utilized a systems-based approach integrating global miRNA profiling of highly and poorly angiogenic tumours, large-scale patient data sets and miRNA (microRNA)–mRNA (messenger RNA) correlation analyses, to identify key miRNAs that are critical for regulating tumour angiogenesis. This unbiased approach led us to identify miR-192 as a key regulator of tumour angiogenesis. In both highly angiogenic ovarian and renal cancer models, we show that miR-192 significantly disrupts the crosstalk between tumour and endothelial cells by targeting two key transcription factors, *EGR1* and *HOXB9*. This led to global downregulation of pro-angiogenic factors. Delivery of miR-192 to tumours using the 1,2-dioleoyl-sn-glycero-3-phosphatidylcholine (DOPC) nanoliposomal platform significantly inhibited tumour angiogenesis, resulting in a much more profound anti-tumour effect compared with that observed with murine anti-VEGF antibody (equivalent to bevacizumab, a clinically approved anti-angiogenic agent). Collectively, these findings provide an understanding of the mechanisms by which miR-192 can regulate tumour angiogenesis and supports its use as an effective therapeutic means of inhibiting tumour angiogenesis.

## Results

### Effect of miR-192 on angiogenesis

**Figure 1:**
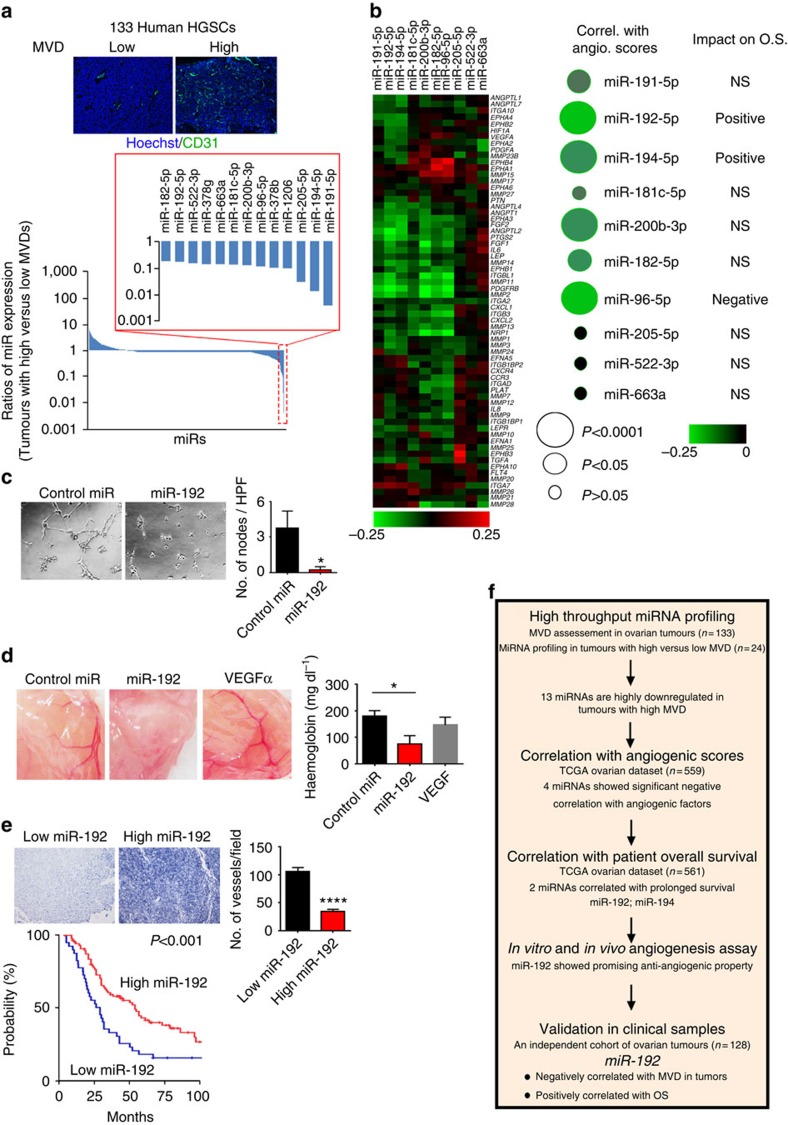
Integrative analyses identified miR-192 as a key player in tumour angiogenesis. (**a**) MiRNA profiling in tumours with very high (>36 vessels per high power field (HPF), *n*=12) or low (<16 vesselsper HPF, *n*=12) microvessel density (MVD). MVD was assessed in high-grade serous ovarian cancers (HGSC) using CD31 staining (*n*=133). Representative images of tumours with high and low MVD are shown. (**b**) Correlation (Correl.) between the levels of angiogenic (angio.) factors and expression of miRNAs that were downregulated in highly angiogenic tumours. Correlation analysis was performed using TCGA ovarian cancer data set (*n*=559, Spearman test). Angiogenesis scores were computed for each individual sample based on the overall relative expression of the pro-angiogenic factors (Supplementary Data 2). The log-rank test was used to determine the association between miRNA expression and OS. (**c**) Tube formation potential of RF24 cells following the exposure to conditioned media collected from SKOV3ip1 cells treated with control miRNA or miR-192 (48 h post transfection). Tube formation potential was assessed 6 h post incubation (*n*=5, Student's *t*-test). (**d**) Representative images (left) and haemoglobin quantification (right) of the *in vivo* matrigel plug assay (*n*=3, Student's *t*-test, 7 days post injection). Matrigel was mixed with VEGF alone (positive control) or conditioned media collected from SKOV3ip1 cells treated with control miRNA or miR-192. (**e**) Correlation between miR-192 expression levels and MVD (Student's *t*-test) or OS (log-rank test) in an independent cohort of human ovarian tumours (*n*=128). MVD was quantified via CD34 staining. (**f**) A flow-chart describing methods used for identifying important anti-angiogenic miRNAs. Scale bar, 100 μm. Bars and error bars represent mean values and the corresponding SEMs (*P<0.05; ****P<0.0001).

**Figure 2:**
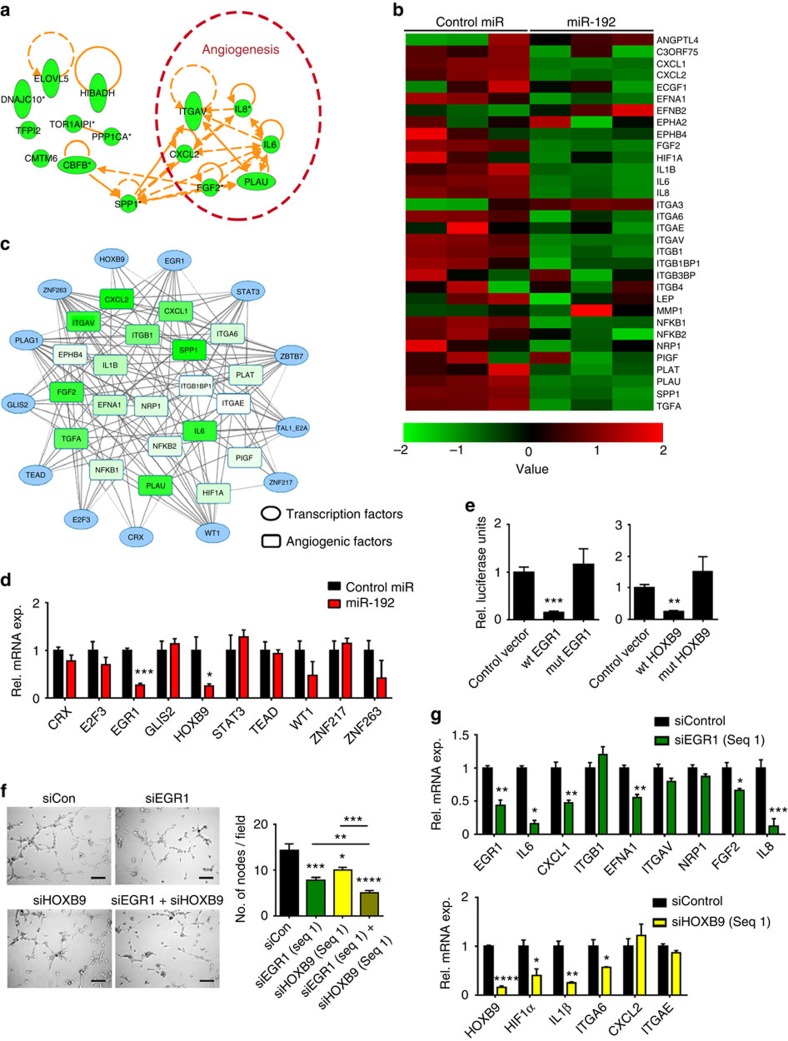
The anti-angiogenic effect of miR-192. (**a**) Top 15 molecules downregulated by miR-192. Whole-genome microarray was performed at 48 h post transfection in SKOV3ip1 cells (*n*=3). (**b**) The effect of miR-192 on pro-angiogenic factors. (**c**) Transcription factors predicted to regulate >5 angiogenic factors and are potential direct targets of miR-192. The lines indicate potential interactions between the transcription factors and the pro-angiogenic genes. (**d**) The effect of miR-192 on the levels of transcription factors that can potentially mediate the broad anti-angiogenic function of miR-192. The mRNA levels of each transcription factor was assessed at 24 h post transfection in SKOV3ip1 cells (*n*=3, Student's *t*-test). (**e**) Relative (Rel.) luciferase activity normalized to empty control for *EGR1* or *HOXB9* 3′-untranslated region (UTR). Mutated constructs have predicted miR-192 binding site deleted. SKOV3ip1 cells were transfected with miR-192 and UTR luciferase constructs using FuGene transfecting reagent, and luciferase assay was performed at 24 h post transfection (*n*=4, Student's *t*-test). (**f**) Tube formation potential was assessed in RF24 cells after exposing to conditioned media collected from SKOV3ip1 cells treated with si*EGR1* and/or si*HOXB9* (48 h post transfection, *n*=5, Student's *t*-test). (**g**) Effect of *EGR1* or *HOXB9* silencing on levels of angiogenic factors. SKOV3ip1 cells were treated with siRNAs and RNA was isolated at 48 h post transfection (*n*=3, Student's *t*-test). Scale bar, 100 μm. All bars and error bars represent mean values and the corresponding s.e.m. (*P<0.05; **P<0.01; ***P<0.001; and ****P<0.0001).

To systematically identify miRNAs that play critical roles in tumour angiogenesis, integrative computational analyses were performed using clinically annotated ovarian tumour samples to identify miRNAs with low endogenous levels that correlate with (1) high microvessel density (Fig. 1a), (2) high levels of angiogenic factors and (3) poor clinical outcome (Fig. 1b). First, human high-grade serous ovarian cancers (HGSC) were assessed for microvessel density (MVD) by CD31 immunohistochemical staining (*n*=133, Fig. 1a). High-throughput miRNA profiling was subsequently performed using Nanostring technology in 24 tumour samples with very high (>36 vessels per high power field (HPF), *n*=12) or low (<16 vessels per HPF, *n*=12) MVD (Supplementary Data 1). All the 24 samples had <25% stromal content, as confirmed by microscopic examination of the tumour sections. For this study, we focused our efforts on identifying miRNAs that are downregulated in tumours with high MVD. From this analysis, 13 miRNAs were found to be decreased by more than 80% (median miRNA expression) in highly angiogenic tumours when compared with poorly angiogenic tumours. We next examined the correlation between tumoral expression of miRNAs and pro-angiogenic factors for these 13 miRNAs in The Cancer Genome Atlas (TCGA) ovarian cancer data set (*n*=559). For each sample in TCGA data set, an angiogenesis score was calculated based on the overall relative expression values for each of the pro-angiogenic factors listed in Supplementary Data 2 (ref. 10). The correlation coefficient between angiogenesis scores and miRNA expression was subsequently computed for each miRNA (Fig. 1b). Among all the miRNAs examined, miR-96, -192, -194 and -200b were found to display the strongest negative correlations with pro-angiogenic factors (*P*<0.0001, Supplementary Table 1). We next assessed the impact of these four miRNAs on patient survival. High tumoral miR-192 and miR-194 correlated with prolonged overall survival (OS; both *P*<0.05). In contrast, tumoral miR-96 expression correlated with poor OS, while no correlation was observed for miR-200b.

To study the roles of miR-192 and miR-194 in tumour angiogenesis, we first examined the expression levels of these miRNAs in endothelial cells isolated from human normal ovaries or HGSCs. No statistically significant differences in miR-192 or miR-194 expression were observed between normal versus tumoral endothelial cells (Supplementary Fig. 1a). We thus reasoned that the effect of these miRNAs on angiogenesis may result from a paracrine effect from tumour cells. To test this hypothesis, we introduced miRNA mimics into SKOV3ip1 ovarian cancer cells, which have low endogenous expression of these two miRNAs (Supplementary Fig. 1b). RF-24, human endothelial cells, were then exposed to conditioned media obtained from control miRNA, miR-192 or miR-194-treated SKOV3ip1 cells (48 h post transfection). Compared with control miRNA treatment, miR-192 treatment resulted in >90% reduction in endothelial tube formation (Fig. 1c), while only a 20% reduction was observed in the miR-194 treatment group (Supplementary Fig. 1c). A similar result was also observed with HeyA8 cells (Supplementary Fig. 1d). Consistent with these findings, miR-192 transfection in SKOV3ip1 and HeyA8 cells resulted in a significant downregulation of several important angiogenic factors including *IL6*, *IL8* and *FGF2* levels (75, 80 and 50% decrease, respectively; Supplementary Fig. 1e), while only a minimal downregulation of these factors was observed following miR-194 treatment (Supplementary Fig. 1f). Incubation of the RF24 cells with conditioned media collected from the SKOV3ip1 cells treated with miR-215, a miRNA closely associated with miR-192, did not significantly inhibit tube formation, demonstrating the unique ability of miR-192 to regulate tumour angiogenesis (Supplementary Fig. 1g). Of note, direct expression of miR-192 in RF-24 cells resulted in modest reduction in their tube formation potential (Supplementary Fig. 1h). This indicates that miR-192 mediates its anti-angiogenic effect mainly from its effect on cancer cells. To further evaluate the impact of miR-192 on tumour angiogenesis, an *in vivo* matrigel plug assay was next performed using conditioned media collected from the SKOV3ip1 cells treated with control miRNA or miR-192. Compared with VEGF (positive control) and conditioned media obtained from control miRNA-treated cells, miR-192 treatment led to substantial reductions in MVD and haemoglobin content (58% decrease, *P*<0.05), a surrogate marker for functional blood flow (Fig. 1d).

To further demonstrate the impact of miR-192 on angiogenesis in clinical samples, an independent set of patient tumours (*n*=128) were used to assess the correlation between miR-192 and tumour MVD. Tumoral miR-192 expression levels showed significant inverse correlation with blood vessel density, with a 68% reduction in MVD in tumours with high levels of miR-192 compared with those with low tumoral levels of miR-192 (percentile cutoff=0.33, *P*<0.0001, Fig. 1e). In this patient cohort, low tumoral miR-192 expression was also found to be significantly associated with worse OS (3.86 versus 6.85 years, log-rank *P*=0.001, Supplementary Table 2), consistent with the results from TCGA ovarian data set. Importantly, tumoral miR-192 expression remained an independent predictor of survival following the multivariate analysis accounting for age, stage, grade and extent of cytoreduction (*P*<0.001, Cox proportional-hazards model). Our systematic analyses thus identified miR-192 as a key player in tumour angiogenesis (Fig. 1f). Indeed, pathway analysis of genes that are negatively associated with miR-192 expression in TCGA ovarian data set also revealed angiogenesis as one of the major pathways affected by this miRNA (Supplementary Table 3).

### MiR-192 mediates global downregulation of angiogenic factors

To identify the dominant angiogenic factors regulated by miR-192, we first examined the correlation between miR-192 and mRNA level of 65 angiogenic factors in TCGA ovarian cancer data set (Supplementary Data 2). *PTGIS* and *IL6* were among the top molecules identified to have the strongest negative correlation with tumoral miR-192 expression (levels increased by 84%, *P*=2.32 × 10^−8^ and 43%, *P*=0.018 in tumours with low miR-192 compared with the ones with high miR-192, respectively; miR-192 percentile cutoff of 0.26 was used for defining high versus low expression). In contrast to *PTGIS* where the level was not altered following miR-192 transfection, the cells treated with miR-192 displayed significantly lower *IL6* mRNA levels compared with the cells treated with control miRNA (45–95% reduction, Supplementary Figs 1e and 2a). However, a luciferase assay using the entire 3′-untranslated region (3′-UTR) of *IL6* revealed the lack of direct binding of miR-192 to *IL6* (Supplementary Fig. 2b).

To systematically examine the molecular mechanisms by which miR-192 mediates its anti-angiogenic effect, a microarray was performed following miR-192 transfection in the SKOV3ip1 cells (Supplementary Data 3). Zinc Finger E-Box Binding Homeobox 2 (*ZEB2*), a known miR-192 target^11^, was used as a positive control for this experiment (Supplementary Fig. 2c). Pathway enrichment analysis using Netwalker^12^ showed that miR-192 significantly alters the angiogenesis pathway (*P*=7.8 × 10^−6^, Supplementary Data 4) and 6 out of the 15 most significantly downregulated genes are pro-angiogenic (Fig. 2a). Further examination of all pro-angiogenic factors that have log_2_ expression levels above 8 (control miRNA-treated cells) in the microarray data set revealed the ability of miR-192 to globally downregulate angiogenic pathways (Fig. 2b). We subsequently hypothesized that miR-192 mediates its global anti-angiogenic effect by targeting transcription factors that regulate multiple angiogenic molecules. Using the list of pro-angiogenic molecules shown in Fig. 2b, transcription factor analysis was performed using Genomatix software at a stringent matrix similarity score cutoff of 0.9. A total of 158 transcription factors were predicted to regulate more than five angiogenic factors with 13 of them predicted to be direct targets of miR-192 by at least four miRNA target prediction algorithms (Supplementary Table 4 and Fig. 2c). We next assessed the ability of miR-192 to target these transcription factors in SKOV3ip1 cells. The levels of *ZBTB7*, *PLAG1* and *TAL1-E2A* were not examined due to their low expression levels in SKOV3ip1 cells. At 48 h following miR-192 transfection, *EGR1* and *HOXB9* mRNA levels were the most downregulated by approximately 75% (Fig. 2d). The downregulation of *EGR1* and *HOXB9* by miR-192 was further confirmed in another ovarian cancer cell line, HeyA8 (Supplementary Fig. 2d). The EGR1 and HOXB9 protein levels were also both significantly downregulated following miR-192 transfection in both the cell lines examined (Supplementary Fig. 2e). Using the data set described in Supplementary Table 2, we further assessed the correlation between miR-192 and EGR1 or HOXB9 protein expression levels in human ovarian epithelial tumours. Tumoral miR-192 expression levels showed significant inverse correlations with EGR1 (*P*<0.001) and HOXB9 (*P*<0.05) protein expression (*n*=98, Supplementary Fig. 2f). Their expression in tumours also correlated with poor overall survival (Supplementary Fig. 2g). To assess whether *EGR1* and *HOXB9* are direct targets of miR-192, luciferase assays using the entire 3′-UTR were performed. As compared with cells transfected with control miRNA, the relative luciferase activity for *EGR1* and *HOXB9* were both significantly reduced by miR-192 (Fig. 2e). For both genes, mutation of the predicted binding site (Supplementary Fig. 2h) abrogated knockdown, indicating that *EGR1* and *HOXB9* are both direct miR-192 targets.

We next examined the effect of *EGR1* and *HOXB9* on angiogenesis. Tube formation assays were performed using RF24 cells exposed to conditioned media collected from either SKOV3ip1 (Fig. 2f) or HeyA8 cells (Supplementary Fig. 2i) following silencing of *EGR1* and/or *HOXB9*. Compared with control short interfering RNA (siRNA), *EGR1* silencing in the SKOV3ip1 cells resulted in 40% decrease in tube formation potential. Silencing of *EGR1* using another siRNA sequence in HeyA8 cells also produced a similar effect (Supplementary Fig. 2i). Exposing RF24 cells to conditioned media collected from *HOXB9* silenced cells also reduced their tube formation potential irrespective of the cell line or siRNA sequence used (Fig. 2f and Supplementary Fig. 2i). Silencing both *EGR1* and *HOXB9* resulted in a further reduction of tube formation potential (Fig. 2f, *P*<0.01 or *P*<0.0001, compared with si*EGR1* or si*HOXB9* treatment alone, respectively). This effect was not fully rescued by replacing *IL6* and *IL8*, two cytokines that are most downregulated by miR-192 treatment, in the conditioned media (Supplementary Fig. 2j). Importantly, we further showed that the observed decrease in tube formation potential upon *EGR1* or *HOXB9* silencing in these cells is not a result of decreased cell viability (Supplementary Fig. 2k).

**Figure 3:**
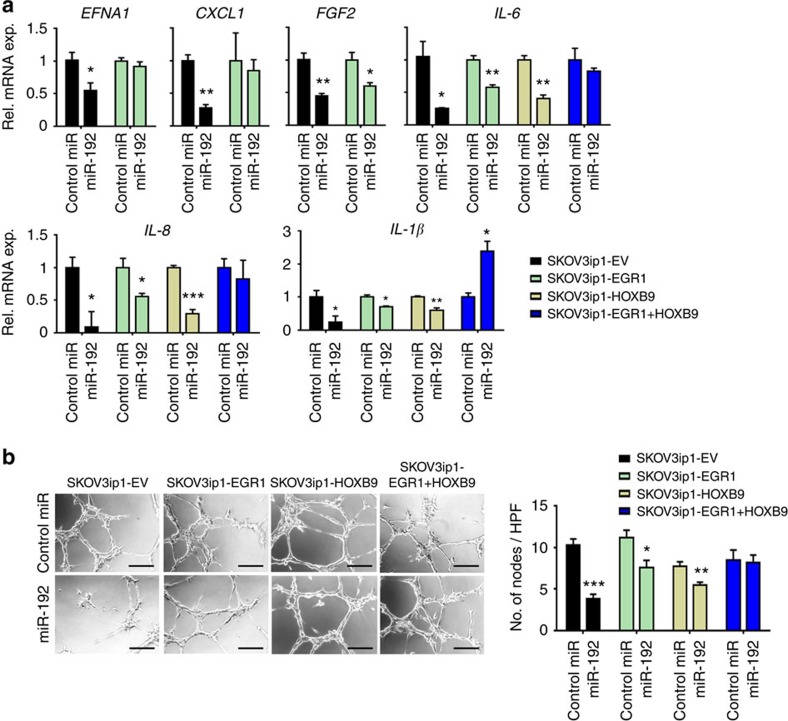
MiR-192 mediates its anti-angiogenic effects through regulating *EGR1* and *HOXB9*. (**a**) SKOV3ip1 cells stably expressing empty vector (EV), *EGR1*, *HOXB9* or *EGR1*+*HOXB9*, were treated with control miRNA or miR-192. Expression levels of pro-angiogenic factors downstream of *EGR1* (*EFNA1*, *CXCL1*, *FGF2*) or downstream of both *EGR1* and *HOXB9* (*IL-6*, *IL-8* and *IL-1β*) were assessed at 48 h following transfection (*n*=3, Student's *t*-test). (**b**) Tube formation potential of RF-24 cells was assessed following incubation with conditioned media collected from SKOV3-EV, *EGR1*, *HOXB9* and *EGR1*+*HOXB9* cells treated with control miRNA or miR-192 (48 h post transfection, *n*=5, Student's *t*-test). Scale bar, 100 μm. High power field, HPF; all the images were taken at × 200 magnification. All the bars and error bars represent mean values and the corresponding s.e.m. (*P<0.05; **P<0.01; and ***P<0.001).

To investigate whether the decrease in angiogenic potential upon *EGR1* or *HOXB9* silencing is a result of the decreased production of pro-angiogenic cytokines, we examined mRNA levels of pro-angiogenic factors that are predicted to be directly regulated by *EGR1* or *HOXB9*. *EGR1* silencing in the SKOV3ip1 cells resulted in significant downregulation of *IL6*, *IL8*, *CXCL1*, *EFNA1* and *FGF2* whereas *HOXB9* silencing mediated downregulation of *HIF1α*, *IL1β* and *ITGA6* (Fig. 2g). Notably, *EGR1* and *HOXB9* were both regulated by miR-192 but had different downstream targets. Similar effects were observed in HeyA8 cells (Supplementary Fig. 2l). We further showed, via chromatin immunoprecipitation (ChIP) assays, that EGR1 and HOXB9 can directly bind to the promoter regions of *IL6* and *EFNA1* (EGR1) and *IL1β* and *ITGA6* (HOXB9; Supplementary Fig. 2m). Next, *EGR1* and *HOXB9* constructs which lack the 3′-UTR component were used to generate cells that are insensitive to miR-192 treatment. Importantly, restoring *EGR1* abrogated the miR-192 induced downregulation of *EFNA1* and *CXCL1.* In contrast, expression of both *EGR1* and *HOXB9* was necessary to completely abrogate the effects of miR-192 on *IL6*, *IL8*, and *IL1β* levels, with a rebound effect observed with *IL1β* (Fig. 3a). This rescue of pro-angiogenic factor expression in *EGR1* and/or *HOXB9* expressing cells resulted in significant abrogation of the anti-angiogenic effects of miR-192. In contrast to empty lentiviral vector (EV)-transduced SKOV3ip1 cells where miR-192 treatment resulted in a 60% reduction in tube formation potential, only 30 and 20% downregulation in tube formation potential was achieved using conditioned media collected from *EGR1*- and *HOXB9*-expressing SKOV3ip1 cells following miR-192 treatment, respectively (Fig. 3b). Dual *EGR1* and *HOXB9* expression resulted in complete rescue of the anti-angiogenic effect of miR-192. Similar findings were also observed in HeyA8 cells (Supplementary Fig. 3). These results indicate that *EGR1* and *HOXB9* are necessary and sufficient to account for the anti-angiogenic effects of this miRNA.

### Upstream regulation of miR-192

Next, we investigated the potential mechanism by which miR-192 is downregulated in tumours. We first correlated miR-192 expression with copy number alteration and promoter methylation in each of the TCGA data sets (21 cancer types). No significant correlation was noted between copy number and expression. However, we observed strong negative correlations between methylation levels of four probes, which target miR-192 directly and overall miR-192 expression levels in multiple cancer types (Supplementary Table 5). Correlation in ovarian cancer was not assessed here as insufficient number of ovarian cancer samples had methylation 450 data available. As an alternative, to assess whether miR-192 expression is indeed regulated by methylation in ovarian cancer, we treated the SKOV3ip1 cells with azacitidine, a methylation inhibitor. Unexpectedly, no rescue of miR-192 level was observed following the azacitidine treatment in the SKOV3ip1 cells (Supplementary Fig. 4a), despite successful demethylation of the miR-192 promoter region (Supplementary Fig. 4b). Minimal rescue of miR-192 levels was observed following the azacitidine treatment in HeyA8 cells (Supplementary Fig. 4a,b).

To examine other potential regulators of miR-192, we used Genomatix software to predict potential transcription factors that can bind to the miR-192 promoter region. Eighty-three potential candidates were identified using a stringent matrix similarity score cutoff of 0.95. Of these genes, *ZEB1*, *ETV1* and *TWIST1* were found to have significant correlations with miR-192 expression in TCGA ovarian database (*n*=559, *P*<0.0001). Upon examining expression levels of these genes and miR-192 in the NCI-60 cell line database^13^, we found *TWIST1* to have the most significant negative correlation with miR-192 expression (Supplementary Fig. 4c). We, thus, hypothesized that *TWIST1*, a transcription factor commonly upregulated in hypoxia, can act as a transcriptional repressor for miR-192. Indeed, when we introduced *TWIST1* into both SKOV3ip1 and HeyA8 cells, miR-192 levels were significantly decreased (Supplementary Fig. 4d). The ability of TWIST1 to bind to the promoter region of miR-192 was further assessed using a ChIP assay with an anti-TWIST1 antibody. Significant fold enrichment in the binding of TWIST1 to the miR-192 promoter region was observed compared with the IgG control (Supplementary Fig. 4e, *P*<0.01). These results indicate the potential role of *TWIST1* in regulating miR-192 in ovarian tumours. Given the role of *TWIST1* in regulating epithelial–mesenchymal transition, we further assessed whether there is a relationship between miR-192 level and epithelial morphology of ovarian tumours. We examined miR-192 expression in mesenchymal versus epithelial subtypes of ovarian tumours using TCGA ovarian data set^14^. We found that *TWIST1* is expressed at a much higher level in the mesenchymal group when compared with the epithelial group (Supplementary Fig. 4f, *P*=2.2e−16). In contrast, miR-192 was expressed at a significantly lower level in the mesenchymal group (*P*=0.02), but the difference in expression between the epithelial and mesenchymal groups was marginal. This indicates that changes in epithelial–mesenchymal transition phenotype may not be the main function of this miRNA. We, thus, reasoned that while *TWIST1* and miR-192 are closely linked with each other, the survival benefit that we observed with miR-192 is not purely a reflection of the difference in *TWIST1* expression.

### *In vivo* effects of miR-192 on ovarian cancer progression

**Figure 4:**
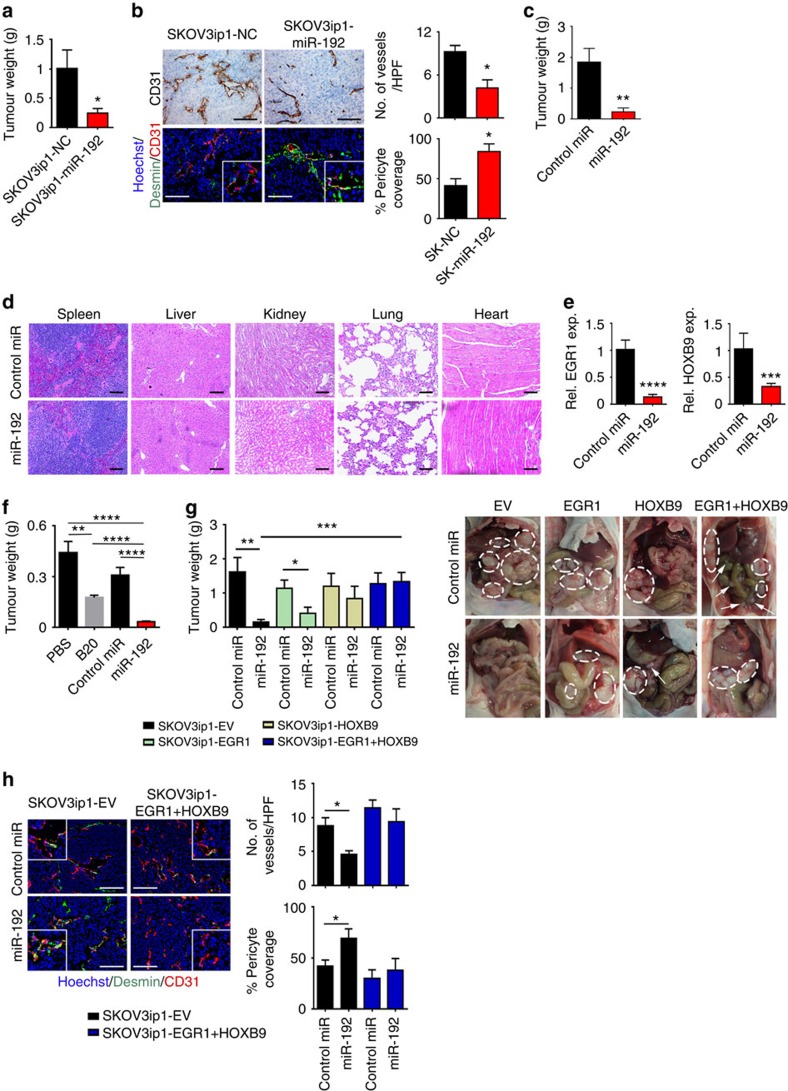
Anti-angiogenic and therapeutic effects of miR-192 in orthotopic mouse models of ovarian cancer. (**a**) Aggregate mass of intraperitoneal implanted SKOV3ip1-NC and SKOV3ip1-miR192 tumours (*n*=10, Student's *t*-test). (**b**) Effect of miR-192 expression on MVD and vessel pericyte coverage in SKOV3ip1 tumours (*n*=5, Student's *t*-test). Higher magnification images are shown in the insets. (**c**) Effect of miR-192-DOPC treatment on tumour weight in SKOV3ip1 tumour-bearing mice (*n*=10, Student's *t*-test). (**d**) Histopathological examination of major organs following 4 weeks of control miRNA-DOPC or miR-192-DOPC therapy (*n*=3). (**e**) Tumoral *EGR1* and *HOXB9* levels following two doses of control miRNA-DOPC or miR-192-DOPC therapies in SKOV3ip1 tumours (*n*=5, Student's *t*-test). (**f**) The effects of B20, control miRNA-DOPC or miR-192-DOPC on tumour burden in mice bearing OVCA-432 tumours (*n*=10, student *t*-test). (**g**) The impact of miR-192-DOPC on tumour burden in orthotopic SKOV3-EV, *EGR1*, *HOXB9* and *EGR1*+*HOXB9* tumours (left, *n*=10, Student's *t*-test). Representative images are shown (right; white circles: tumours). (**h**) Representative images of CD31 (red), desmin (green) and nuclei visualized with Hoechst 33342 (blue) in SKOV3ip1-EV and *EGR1*+*HOXB9* tumours. Higher magnification images are shown in the insets. Bar graphs (right) show the quantitative analyses of CD31 and desmin staining (*n*=5, Student's *t*-test). Bars and error bars represent mean values and the corresponding s.e.m. (*P<0.05; **P<0.01; ***P<0.001; and ****P<0.0001).

Given the anti-angiogenic property of miR-192, we next examined the effect of miR-192 on tumour progression in orthotopic mouse models of ovarian cancer. We first introduced lentiviral vectors expressing control miRNA (SKOV3ip1-NC) or miR-192 (SKOV3ip1-miR-192) into SKOV3ip1 cells. Compared with SKOV3ip1-NC cells, SKOV3ip1-miR-192 cells exhibit a significant increase in miR-192 level (Supplementary Fig. 5a). Similar to the SKOV3ip1 cells treated with miR-192 mimics (Supplementary Fig. 1d), the RF24 cells treated with conditioned media collected from SKOV3ip1-miR-192 cells showed significantly reduced tube formation potential compared with that of SKOV3ip1-NC cells (Supplementary Fig. 5b). We subsequently injected SKOV3ip1-NC or SKOV3ip1-miR-192 cells into the peritoneal cavity of athymic nude mice (*n*=10 per group). Tumour burden was assessed 4 weeks following the cell injection. Mice bearing SKOV3ip1-miR-192 tumours showed a 70% decrease in tumour burden (*P*<0.05, Fig. 4a) compared with mice in the SKOV3ip1-NC group. The number of tumour nodules was also decreased in mice bearing miR-192-expressing tumours (Supplementary Fig. 5c). Compared with SKOV3ip1-NC tumours, SKOV3ip1-miR-192 tumours showed a significant 55% reduction in MVD (*P*<0.05), with a 50% increase in the percentage of blood vessels covered with pericytes (*P*<0.05, Fig. 4b). This further corroborates the paracrine effect of tumoral miR-192 expression on blood vessel maturation. Notably, stable transduction of SKOV3ip1 with miR-192 had no effect on the growth rate compared with cells transduced with control miRNA *in vitro* (Supplementary Fig. 5d). These findings further point to the role of tumour microenvironment in mediating the anti-tumour effects of miR-192.

Having established the anti-tumour effect of miR-192 *in vivo*, we next tested the feasibility of using miR-192 therapeutically to treat ovarian cancer. MiRNAs were delivered via intraperitoneal injection twice weekly using a well-characterized nanoparticle platform, neutral DOPC nanoliposomes^15^,^16^,^17^. The SKOV3ip1 cells were injected into the peritoneal cavity of mice (*n*=10 per group). There were two treatment groups: (1) non-targeting control (NC) miRNA-DOPC or (2) miR-192-DOPC. After 4 weeks of therapy, the animals were killed and necropsies were performed. Significant decreases in tumour weight (85%, *P*<0.01), number of tumour nodules (80%, *P*<0.01) and volume of ascites (95%, *P*<0.05) were observed following miR-192-DOPC treatment when compared with mice treated with NC miRNA-DOPC (Fig. 4c, Supplementary Fig. 5e). No group showed decreased body weight, a major indicator of toxicity (Supplementary Fig. 5f). Moreover, histopathological examination of major organs following 4 weeks of miR-192-DOPC therapy revealed no abnormalities (Fig. 4d). Importantly, significant reductions in *EGR1* and *HOXB9* levels were observed in tumours following two doses of miR-192-DOPC treatments, compared with tumours treated with control miRNA-DOPC (Fig. 4e). Next, a survival experiment was carried out to assess the impact of miR-192 on survival in mice (*n*=7). Mice bearing SKOV3ip1 tumours were treated with control miR or miR-192 containing DOPC nanoliposomes. The mice were killed individually once they became moribund, and a Kaplan–Meier curve was used to analyse the survival difference between the different treatment groups. We showed that miR-192 significantly enhanced survival (*P*=0.009, Supplementary Fig. 5g).

Given the ability of miR-192 to modulate tumour angiogenesis, we next assessed the feasibility of treating ovarian tumours with combined therapy of miR-192 and topotecan, a chemotherapeutic agent with anti-angiogenic activity^18^. The mice were treated with miRNAs-DOPC twice weekly as described above and topotecan was administered once weekly (intravenously, 5 mg kg^−1^) starting 7 days after tumour cell implantation. The mice were killed after 4 weeks of therapy. Similar to the results obtained previously, miR-192-DOPC treatment reduced the tumour burden by approximately 85% (SKOV3ip1 model, Supplementary Fig. 5h). Compared with NC miRNA-DOPC alone, the mice treated with topotecan and NC miRNA-DOPC resulted in a 50% reduction in tumour burden. Combination treatment of miR-192 and topotecan resulted in a 40% further reduction in tumour weight compared with mice treated with NC miRNA-DOPC plus topotecan. We next sought to compare the anti-angiogenic and anti-tumour effect between miR-192 and murine anti-VEGF antibody, B20-4.1.1 (equivalent to bevacizumab, a clinically used anti-VEGF agent for the treatment of multiple cancer types). OVCA-432 high-grade serous ovarian cancer cells were injected into the peritoneal cavity of mice (*n*=10 per group) and all the treatments started after tumours had been established (3 weeks following the tumour cell injection). There were four treatment groups: (1) Phosphate-buffered saline (PBS), (2) B20-4.1.1, (3) NC miRNA-DOPC and (4) miR-192-DOPC. All the mice were killed after 3 weeks of therapy. As expected, B20-4.1.1 treatment resulted in a significant reduction in tumour burden (60% reduction compared with PBS treatment, *P*<0.01, Fig. 4f). Notably, miR-192-DOPC therapy induced an even more profound tumour regression effect, resulting in >90% reduction in tumour weight compared with PBS treatment (*P*<0.0001). As miR-192 expression has been previously reported to be associated with VEGF levels^19^, we assessed both miR-192 and miR-194 levels in tumours treated with B20. As expected, there was a modest increase in miR-192 level in B20-treated tumours when compared with the PBS control group, although the difference was not statistically significant (Supplementary Fig. 5i). This degree of increase in miR-192 level was significantly lower than that observed from miR-192-DOPC treatment (Supplementary Fig. 5j). Importantly, miR-192-DOPC treatment resulted in significant reduction in blood vessel density as well as increased pericyte coverage of residual blood vessels (Supplementary Fig. 5k). We showed that the ability of miR-192 to reduce blood vessel density was more profound than that observed with the B20 treatment. Importantly, B20 treatment did not affect the percentage of pericyte coverage of residual blood vessels. We further showed that miR-192-DOPC treatment resulted in a significant increase in the number of apoptotic cells in tumours (*P*<0.001, Supplementary Fig. 5l). Collectively, these results indicate that significant therapeutic benefit can be derived from miR-192-mediated inhibition of multiple pro-angiogenic pathways simultaneously.

We next assessed whether the anti-angiogenic effect of miR-192 can be abrogated by *EGR1* and/or *HOXB9* expression in SKOV3ip1 tumours. *EGR1* and *HOXB9* constructs that lack the 3′-UTR component were used to generate cells that are insensitive to miR-192 treatment. SKOV3ip1-empty vector (EV), SKOV3ip1-*EGR1*, SKOV3ip1-*HOXB9* and SKOV3ip1-*EGR1*+*HOXB9* cells were injected into the peritoneal cavity of mice. The mice were then treated with control miRNA-DOPC or miR-192-DOPC twice weekly for 4 weeks. In contrast to the SKOV3ip1-EV tumours where miR-192 treatment resulted in a 90% reduction in tumour burden, only 65 and 30% reduction in tumour burden was observed for tumours expressing *EGR1* and *HOXB9*, respectively (Fig. 4g). The expression of both *EGR1* and *HOXB9* in tumours completely abrogated the anti-tumour effect of miR-192. Only minimal decreases in MVD and increases in the extent of pericyte coverage of residual blood vessels were observed in tumours expressing both *EGR1* and *HOXB9* after the miR-192 treatments (Fig. 4h), emphasizing the key roles *EGR1* and *HOXB9* play in mediating the anti-angiogenic effects of miR-192. We further confirmed that while *EGR1* and *HOXB9* expression decreased the extent of pericyte coverage around the blood vessels, their expression has minimal effect on desmin levels in pericytes or pericyte-like cells (Fig. 4h and Supplementary Fig. 5m). Importantly, in SKOV3ip1-EV tumours, tumour vasculature following the miR-192 treatment was significantly less permeable than the control miRNA group (Supplementary Fig. 5n). In contrast, minimal difference in vessel permeability was observed between SKOV3ip1-*EGR1*+*HOXB9* tumours treated with control miRNA and miR-192.

### Effects of miR-192 on angiogenesis in renal cancer

**Figure 5:**
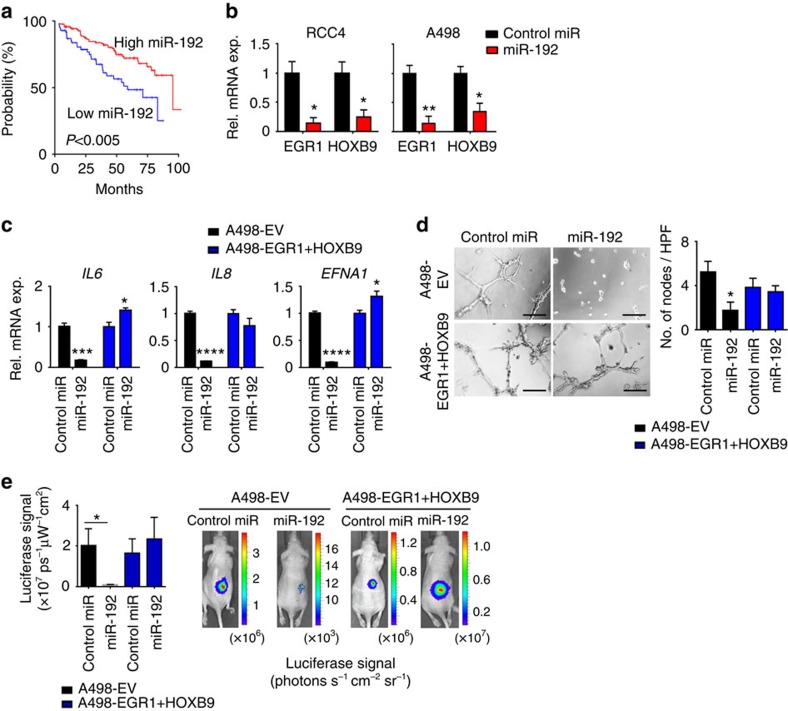
The anti-angiogenic and antitumour effects of miR-192 in renal tumours. (**a**) Kaplan–Meier plot for OS based on tumoral miR-192 expression for patients with renal tumours in TCGA KIRC data set (*n*=413, log-rank test). (**b**) *EGR1* and *HOXB9* levels in RCC4 and A498 cells at 24 h following control miRNA or miR-192 treatment (*n*=3, Student's *t*-test). (**c**) The effect of miR-192 on levels of key angiogenic factors in A498-EV and A498-*EGR1*+*HOXB9* cells (*n*=3, Student's *t*-test). *IL6*, *IL8* and *EFNA1* are among the angiogenic factors that are most significantly downregulated by miR-192 in A498 cells. (**d**) Tube formation potential of RF24 cells following incubation with conditioned media collected from control miRNA or miR-192 treated A498-EV or A498-*EGR1*+*HOXB9* cells. Bar graph (right) shows the quantitative analyses of number of nodes per HPF (*n*=5, Student's *t*-test). (**e**) The effect of miR-192 on tumour burden in mice bearing luciferase-labelled A498-EV or A498-*EGR1*+*HOXB9* tumours. The graph (left) shows quantitative assessment of the total absolute luciferase signal (photons s^−1^ cm^−2^ sr^−1^, *n*=10, student *t*-test). Representative images from each treatment group are shown (right). Scale bar, 100 μm. All bars and error bars represent mean values and the corresponding SEMs. (*P<0.05; **P<0.01; ***P<0.001; and ****P<0.0001).

To assess whether miR-192 can also affect tumour angiogenesis in other cancer types, we examined the level of miR-192 in tumours across 19 cancer types using TCGA data sets (Supplementary Fig. 6a). Since clear-cell renal carcinomas are highly angiogenic^20^, and anti-angiogenic agents are the mainstay of treatment for kidney cancer, we hypothesized that miR-192 treatment may show therapeutic benefit in renal cancer patients with low endogenous tumoral miR-192 expression. Indeed, renal cancers were found to have a large dynamic range of miR-192 expression (Supplementary Fig. 6a), and patients with tumours expressing low levels of miR-192 had significantly worse OS (median OS of 69 versus 147 months for tumours with low versus high miR-192 expression, respectively, *P*<0.005; percentile cutoff=0.29; Fig. 5a).

We introduced miR-192 mimics into two renal cancer cell lines, RCC4 and A498. Similar to ovarian cancer, miR-192 treatment mediated a robust downregulation of *EGR1* and *HOXB9* levels (Fig. 5b) and significant decreases in several angiogenic factors including *IL6*, *IL8* and *EFNA1* (Fig. 5c); consistent with the pattern observed after si*EGR1*/si*HOXB9* treatment (Supplementary Fig. 6b,c). Exposing the RF24 cells to conditioned media collected from RCC4 or A498 cells treated with miR-192 resulted in 50–70% decrease in tube formation potential when compared with control miRNA treatment (Fig. 5d, Supplementary Fig. 6d). This miR-192-mediated anti-angiogenic effect was significantly reduced following the rescue of *EGR1* and/or *HOXB9* levels by lentiviral transduction in these renal cancer cells (Fig. 5c,d, Supplementary Fig. 6d).

To assess the therapeutic effect of miR-192 in renal cancer, we injected A498 renal cancer cells expressing an EV or both *EGR1* and *HOXB9* (A498-EV and A498-*EGR1*+*HOXB9*, respectively) into the subcapsular space in the kidneys of mice. All the cells were luciferase-labelled to enable the assessment of tumour burden. Starting 7 days following the cell implantation, the mice were treated with DOPC nanoliposome-delivered control miRNA or miR-192 twice a week. MiR-192 treatment resulted in a >95% decrease in tumour burden in mice bearing A498-EV tumours (Fig. 5e). In contrast, the tumours expressing EGR1 and HOXB9 proteins failed to respond to miR-192 therapy, again demonstrating the central role of these two proteins in miR-192-mediated anti-tumour effect. Collectively, both the *in vitro* and *in vivo* results shown here were consistent with those observed in ovarian cancer models, indicating the broad implications of modulating miR-192 in different cancer types.

## Discussion

**Figure 6:**
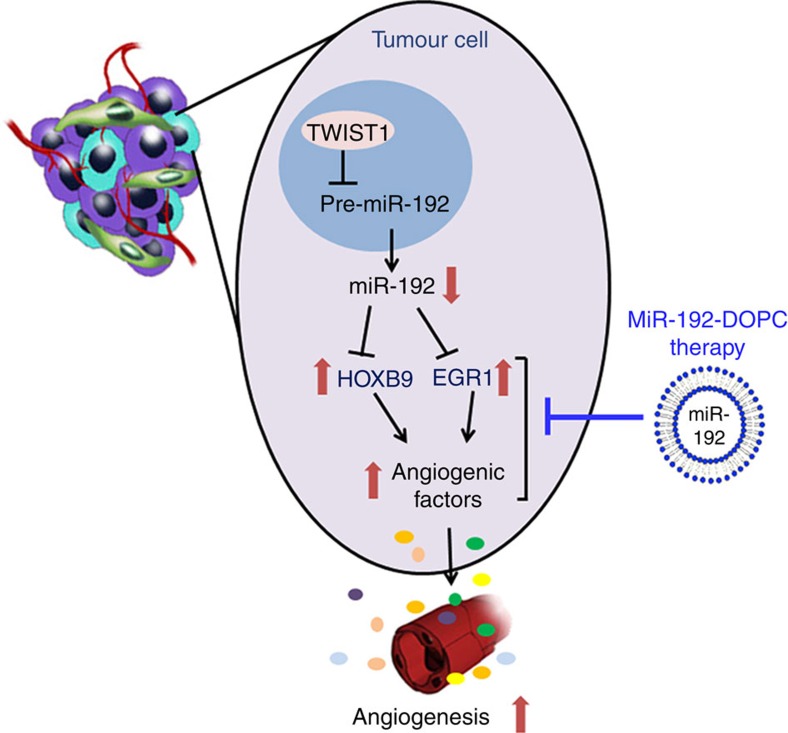
Schematic representation of mechanisms by which miR-192 mediates its anti-angiogenic function. On the basis of findings described in the manuscript, *TWIST1* downregulates miR-192 levels in cancer cells, leading to increased *HOXB9* and *EGR1* levels. These two transcription factors, in turn, lead to increased levels of multiple pro-angiogenic factors and increased tumour angiogenesis. Systemic delivery of miR-192 using DOPC nanoliposomes represents a potent means of blocking tumour angiogenesis and reducing tumour growth.

Using an integration of systems-based bioinformatics coupled with experimental models, we rigorously document here the central role of miR-192 in regulating tumour angiogenesis. To date, no other miRNA has been identified to have such a global effect on angiogenesis. We demonstrated that the potent anti-angiogenic effect of miR-192 stems from its ability to globally downregulate angiogenic pathways in cancer cells through regulating two key transcription factors, *EGR1* and *HOXB9* (Fig. 6). Using the DOPC nanoliposomal platform, which is currently being tested in clinical trials, we showed that miR-192 can mediate potent anti-angiogenic and anti-tumour effects in multiple orthotopic mouse models of ovarian and renal cancer. The anti-angiogenic and anti-tumour effects of miR-192 were found to be much more robust than that achieved with anti-VEGF antibody. As upregulation of the compensatory pro-angiogenic signalling cascades has been shown to play a crucial role in resistance to anti-angiogenic therapy, our finding of the global anti-angiogenic effect of miR-192 represents a promising new approach for targeting tumour angiogenesis.

Although the downregulation of miR-192 has been previously observed in multiple cancer types^21^,^22^,^23^,^24^,^25^, its clinical relevance and the feasibility of using it as a therapeutic agent are currently unknown. Here, using large-scale patient data sets, we demonstrated that decreased tumoral miR-192 levels are associated with increased angiogenesis and poor overall survival in patients with high-grade serous ovarian or renal clear cell carcinomas. We further presented several lines of pre-clinical evidence demonstrating the marked therapeutic potential of miR-192 in ovarian and renal cancer models without causing toxicity in normal organs. To date, the biological function of miR-192 has been reported predominantly in the areas of cell survival^26^ and metastasis through direct targeting of *MDM2*, *TYMS*, *SIP1* or *ZEB2* (refs 21, 24, 27, 28, 29). While its effect on these genes could contribute partially to its anti-tumour activity, recent studies that used systematic translational approaches to identify key microRNAs important for metastasis did not identify miR-192 to be the top regulator for cancer metastasis^30^,^31^,^32^. In contrast, the miR-200 family, which has been reported to play key roles in angiogenesis^16^ and metastasis^33^, was identified in both our angiogenesis-specific analysis as well as the metastasis focused analysis reported by Mudduluru *et al*.^30^ The investigation of the specific role of miR-192 in regulating tumour angiogenesis is thus warranted. Previous reports have documented the ability of miR-192 to negatively regulate factors such as *VEGFA*^34^ or *ICAM1* (ref. 35) leading to inhibition of metastatic colonization of tumour cells. However, the mechanism by which this miRNA mediates its broad anti-angiogenic effect was not established. We report here that *EGR1* and *HOXB9* transcription factors are responsible for mediating the broad anti-angiogenic function of miR-192, since restoration of *EGR1* and *HOXB9* completely rescued this effect. Importantly, we showed that miR-192 is able to mediate profound anti-angiogenic and anti-tumour effects *in vivo*, independent of its impact on cancer cell growth.

The central role of *EGR1* and *HOXB9* in tumour angiogenesis has recently been recognized. For instance, *EGR1* was shown to be important in mediating angiopoietin-1-induced endothelial cell proliferation, migration and differentiation^36^. Both these transcription factors have been reported to bind directly to the promoter regions of several angiogenic factors, such as *IL8*, *VEGF* and *FGF2*, resulting in increased production of these cytokines^37^,^38^,^39^,^40^,^41^. Through integrative analyses in this study, we uncovered the new roles of *EGR1* and *HOXB9* in regulating other important angiogenic factors, including *CXCL1*, *EFNA*, *IL6* and *IL1β*. Importantly, *EGR1* and *HOXB9* have both been shown to correlate with poor patient survival in multiple cancer types^42^,^43^,^44^,^45^,^46^. The identification of miR-192 as a common upstream regulator of these two key transcription factors is thus of high importance given that to date, mechanisms of *EGR1* and *HOXB9* regulation are still largely unknown and no effective therapeutic means has been developed to regulate their expression.

Given the ability of miR-192 to globally downregulate the angiogenic pathways, it provides a central node to potently block tumour angiogenesis. The ovarian and renal cancers are both highly angiogenic cancer types, with high miR-192 levels correlating with significantly improved patient survival. As such, it is anticipated that patients whose tumours have low miR-192 expression would greatly benefit from miR-192 therapy. Collectively, our data provide an important advance in understanding the importance and mechanism of miR-192 in regulating tumour angiogenesis. Our complete understanding of this mechanistic pathway will open rational avenues for therapeutic interventions and novel combination therapies for cancer patients.

## Methods

### Cell lines and cell growth assays

All ovarian cancer cell lines (SKOV3ip1, HeyA8, OVCAR-8, OVCA-432) were maintained in RPMI 1640 media supplemented with 15% fetal bovine serum (FBS) and 0.1% gentamicin sulfate (GeminibBioproducts, Calabases, CA, USA; refs 47, 48). The A498 and RCC4 cell lines were kindly provided by Dr Eric Jonasch (M.D. Anderson Cancer Center) and were maintained in DMEM-high glucose supplemented with 10% FBS and 0.1% gentamicin. The RF24 cells were maintained in MEM supplemented with 10% FBS, sodium pyruvate, MEM vitamins, L-glutamine, and MEM non-essential amino acids. 10T1/2 pericyte-like cells were maintained in DMEM-high glucose supplemented with 10% FBS and 0.1% gentamicin. All the cell lines were routinely tested to confirm the absence of Mycoplasma. Cell lines were validated by Short Tandem Repeat (STR) DNA fingerprinting using the Promega 16 High Sensitivity STR Kit. The STR profiles were compared with online search databases (DSMZ/ATCC/JCRB/RIKEN) of 2,455 known profiles; along with the MD Anderson Characterized Cell Line Core database of 2,556 known profiles. The STR profiles matched known DNA fingerprints or were unique. All the *in vitro* experiments were conducted with 60–80% confluent cultures. For cell growth assays, the cells were plated in six-well plates in triplicate and were counted each day for 6 days using a Beckman Coulter cell counter.

### TCGA data exploratory analyses

We used clinically annotated data from TCGA obtained from the Open-Access and Controlled-Access tiers of the TCGA Data Portal ( http://tcga-data.nci.nih.gov/tcga/findArchives.htm) with NIH approval. The expression data for all the miRNAs were obtained from Agilent miRNA microarrays and Illumina miRNA-Seq data sets. For the miRNA-Seq data, we derived the ‘reads_per_million_miRNA_mapped' values for mature forms for each miRNA from the ‘isoform_quantification' files. For the miRNA-Seq data sets, the following MIMAT numbers were analysed: MIMAT0000259 (hsa-miR-182-5p), MIMAT0000222 (hsa-miR-192-5p), MIMAT0002868 (hsa-miR-522-3p), MIMAT0018937 (hsa-miR-378g), MIMAT0003326 (hsa-miR-663a), MIMAT0000258 (hsa-miR-181c-5p), MIMAT0000318 (hsa-miR-200b-3p), MIMAT0000095 (hsa-miR-96-5p), MIMAT0014999 (hsa-miR-378b), MIMAT0005870 (hsa-miR-1206), MIMAT0000266 (hsa-miR-205-5p), MIMAT0000460 (hsa-miR-194-5p) and MIMAT0000440 (hsa-miR-191-5p). The analyses were carried out in R statistical environment (version 3.0.1) ( http:///www.r-project.org/). All the tests were two-sided and considered statistically significant at the 0.05 level. Clinical associations were used to prioritize the 13 anti-angiogenic miRNAs for further investigation (Fig. 1). Kaplan–Meier curves were plotted for all the data sets. For each miRNA, the tumour samples were dichotomized into high and low miRNA expression groups at percentile cutoffs between 0.25 and 0.75 with a step of 0.01. The optimal cutoff percentile (as determined by the lowest *P* value) was identified for each miRNA. For each miRNA, we tested whether a log-rank test applied at any cut-point would yield a nominal *P* value <0.05; this flagged three miRNAs for further investigation.

For the miRNA-angiogenic factor correlation study, we examined the expression levels of all the previously reported pro-angiogenic factors in the TCGA ovarian data set (Agilent miRNA microarray and Agilent mRNA data sets were used). The list of pro-angiogenic factors examined is presented in Supplementary Data 2. We first checked and selected those pro-angiogenic genes that are consistently expressed in TCGA samples. A ‘pro-angiogenic' signature score was subsequently computed for each TCGA sample using the formulae below (equation (1))^10^:


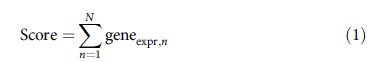


Here, *N* is the number of genes in the list, the parameter gene_expr,*n*_ for a sample is 1 if the sample has a level of gene *n* above the median for all the TCGA samples and −1 otherwise. The list of samples used for this analysis is presented in Supplementary Table 6. The median expression of each angiogenic factor is included in Supplementary Data 2. The correlation between the angiogenesis score and miRNA expression was subsequently assessed for each miRNA for all the samples (Spearman rank correlation). The contrasts between the angiogenic score and component miRNA expression values were also used for filtering miRNAs for follow-up; nominal *P* values for our best four miRNAs were <0.0001 (Fig. 1).

For the methylation analyses, we examined all the tumour tissue types in TCGA that have methylation 450 data available. After eliminating the data from replicate samples, we plotted miR-192 expression against the beta value for each of the associated methylation probes (cg02258444-11-64658622, cg09349409-11-64658765, cg18262830-11-64658819 and cg27083891-11-64658726). We subsequently performed Spearman correlation analyses between miR-192 expression and the beta values for each probe.

### Effect of miR-192 expression on mesenchymal phenotype

Agilent 244K gene expression data and miRNA-seq expression data were obtained from the open-access and controlled-access tiers of the TCGA data portal, with NIH approval. The ovarian cancer tumour subtype information was obtained using a previously established protocol^14^. The expression of miR-192 and TWIST1 were compared between the mesenchymal and epithelial groups using the Wilcoxon rank-sum test.

### *In vitro* miRNA and siRNA transfection

The cells were transfected with 100 nM of specified siRNAs using RNAiMax reagent (Invitrogen) at 3 μl reagent: 1 μg siRNA ratio^49^. The cells were treated with siRNAs for 4 h in serum-free media before incubation in fresh complete media for the specified time frame. All the siRNA sequences are listed in Supplementary Table 7. For miRNA, reverse transfection technique was used^16^. The cells were transfected with 40 nM miRNA in media containing 10% FBS and the media were changed 4 h following transfections to minimize toxicity. All the siRNAs were obtained from Sigma and miRNAs were obtained from Life Technologies. MiRVana miRNA mimic negative control #1 (catalogue number 4464061, ThermoFisher Scientific) was used as the negative control.

### Quantitative reverse transcription polymerase chain reaction

RNA was isolated from the cells or tumours using Direct-zol RNA miniprep kit (Zymo Research) according to the manufacturer's protocol. cDNA was synthesized using a Verso cDNA kit (Thermo Scientific) as per manufacturer's instructions. Quantitative reverse transcription PCR (RT–PCR) was performed on a 7500 Fast Real-Time PCR System (Applied Biosystems)^48^. All primer sequences for mRNA detection are listed in Supplementary Table 8. β-actin mRNA was used as the normalizing control. For miRNA detection, Ambion assay probe sets (Life technologies) were used according to the manufacturer's protocol. RNU44 and RNU6B were used as the normalizing control.

### Cell apoptosis assay

Cell apoptosis assay was performed using the Annexin V/propidium iodide (Invitrogen) staining kit according to the manufacturer's instructions. Cells were stained with fluorescein isothiocyanate-conjugated Annexin V and propidium iodide for 30 min and were analysed by flow cytometry to determine the percentage of apoptotic cells.

### Methylation-specific PCR

MethPrimer software ( http://www.urogene.org/methprimer/) was used for design of methylation-specific primers for miR-192 (Supplementary Table 8). Total DNA was isolated from cells using the DNeasy blood and tissue kit (Qiagen) as per the manufacturer's protocol, followed by treatment with bisulphite using a methylation kit (EZ DNA Methylation-Direct; Zymo Research, Orange, CA). Using real-time PCR, as described above, quantification of methylation was performed by comparing the ratios of unmethylated/methylated levels in the control sample and the samples treated with 5-azacytidine (Sigma).

### 3′-UTR assay

GoClone pLightSwitch *IL-6*, *EGR1* and *HOXB9* 3′-UTR luciferase reporter constructs were obtained from SwitchGear genomics (Menlo Park, CA, USA). For *EGR1* and *HOXB9*, mutated constructs were also synthesized with the predicted miR-192 binding sites deleted (Supplementary Fig. 2h). The SKOV3ip1 cells were transfected with FuGENE HD reagent in a 96-well plate with control miRNA or miR-192 (40 nM) along with the 3′-UTR reporter constructs, and Cypridina TK control construct (pTK-Cluc) as per the manufacturer's protocol^50^. After 24 h of transfection, luciferase activity was obtained using LightSwitch Dual Luciferase assay kits. All the assays were performed in quadruplicate.

### Immunoblotting

Protein lysates were prepared from cultured cells using RIPA buffer supplemented with protease and phosphatase inhibitors^51^. The protein concentrations were determined using a BCA Protein Assay Reagent kit (Pierce Biotechnology, Rockford, IL, USA). The lysates were loaded and separated on 10% SDS–polyacrylamide gels. Nitrocellulose membranes were subsequently probed with primary antibodies against EGR1 (1:1,000, Cell Signaling, Catalogue number 4153), HOXB9 (1:1,000, Abcam, Catalogue number ab66765) and β-actin (1:5,000, Sigma, Catalogue number A5316) in 5% BSA in TBS-T overnight at 4 °C. After washing with TBS-T, the membranes were incubated with horseradish peroxidase (HRP)-conjugated secondary antibodies (1:2,000, GE Healthcare, Catalogue numbers NA931 and NA934) for 2 h. HRP was visualized using an enhanced chemiluminescence detection kit (PerkinElmer, Catalogue number NEL104001EA). Full scans of important western blots are provided in Supplementary Fig. 7.

### ChIP assay

ChIP assays were performed using ChIP-IT Express Kit (Active Motif) as per the manufacturer's instructions. Briefly, crosslinked cells were collected, lysed, sonicated and subjected to immunoprecipitation with TWIST1 (Abcam, Catalogue number Ab50887), EGR1 (Cell Signaling, Catalogue number 4153), HOXB9 (Abnova, Catalogue number H00003219-M05) or IgG isotype control (Millipore, Catalogue number 12–371B for mouse IgG and Cell Signaling, Catalogue number 2729, for rabbit IgG) antibodies. Immunocomplexes were collected using protein A/G agarose magnetic beads and were eluted. PCR-based quantification of fold enrichment in TWIST1, EGR1 or HOXB9 binding to miR-192, *IL6*, *EFNA1*, *IL1β* or *ITGA6* promoter regions was subsequently performed.

### Liposomal nanoparticle preparation

MiRNA for *in vivo* delivery were incorporated into DOPC liposomes^49^,^52^. DOPC and miRNA were mixed in the presence of excess tertiary butanol at a ratio of 1:10 (w/w) miRNA/DOPC. Tween 20 was added to the mixture in a ratio of 1:19 Tween 20:miRNA/DOPC. The mixture was then vortexed, frozen in an acetone/dry ice bath and lyophilized. Before *in vivo* administration, this preparation was hydrated with PBS at room temperature (5 μg miRNA per 200 μl).

### Orthotopic *in vivo* models of ovarian and renal cancer

Female athymic nude mice (8–12 weeks old) were obtained from the National Cancer Institute, Frederick Cancer Research and Development Center (Frederick, MD, USA). All the mouse studies were approved and supervised by the M.D. Anderson Cancer Center Institutional Animal Care and Use Committee. For the therapeutic experiments, 10 mice were assigned per treatment group. This sample size gave 80% power to detect a 50% reduction in tumour weight with 95% confidence. To establish intraperitoneal ovarian tumours, cells were injected into the peritoneal cavity (intraperitoneally) at a concentration of 5–15 × 10^6^ cells ml^−1^ (200 μl per injection). For the renal cancer model, renal cancer cells were injected into the renal subcapsular space in the right kidney (0.3 million cells in 30 μl HBSS). For therapeutic tumour growth inhibition experiments, all the mice were treated with miRNA-DOPC (5 μg, intraperitoneally) twice a week beginning at 1 week following the tumour cell injection. For tumour regression experiments, the mice received miRNA-DOPC treatments (5 μg, intraperitoneally) twice a week starting on day 21 following the tumour cell injection. Luciferase imaging was used to confirm tumour establishment before initiation of the treatments^16^. Anti-VEGF B20–4.1.1 antibodies were administered intraperitoneally twice a week (5 mg kg^−1^, Genetech) and topotecan was administered intravenously once a week (5 mg kg^−1^, Sigma). The mice (*n*=10 per group) were monitored for adverse effects and the tumours were harvested after 3–4 weeks of therapy or when any of the mice began to appear moribund. To examine the vessel permeability and structure, three mice per treatment group received FITC-dextran (100 μl, 10 mg ml^−1^, 2,000,000 MW; Sigma) before being killed. Ten minutes following the dextran injection, the mice were perfused through the left ventricle with 4% paraformaldehyde in PBS^16^. The mouse weight, tumour weight and the number/location of tumour nodules were recorded. The tissues were fixed in 10% formalin or embedded in Tissue-Tek optimal cutting temperature formulation for subsequent sectioning^16^,^49^. For the survival experiment, mice bearing SKOV3ip1 tumours were treated with control miRNA-DOPC or miR-192-DOPC twice a week starting at 1 week following the tumour cell injection. The mice were killed individually once they became moribund. A Kaplan–Meier curve was used to analyse the survival difference between control and treatment groups.

### *In vivo* matrigel plug assay

After transfecting the SKOV3ip1 cells with either control miRNA or miR-192 (40 nM), cells were exposed to serum-free media for 48 h. We then collected the supernatants and centrifuged them to remove cells. The conditioned media were then mixed with phenol-red-free Matrigel (2:3 proportion, total 0.5 ml; BD Biosciences). The mixture was then injected into each mouse subcutaneously (*n*=3 per group)^16^. VEGF (0.25 nM, R & D Systems) was used as a positive control. The mice were killed on day 8 and matrigel plug was examined for haemoglobin content using the QuantiChrom hemoglobin assay kit as per the manufacturer's protocol (BioAssay Systems).

### High-throughput miRNA profiling

Total RNA from human ovarian tumours was extracted using mirVana RNA isolation labeling kit (Ambion) according to the manufacturer's protocol. RNA purity was assessed by a Nanodrop spectrophotometric measurement (Thermo Scientific) of the OD260/280 ratio with acceptable values falling between 1.9 and 2.1. RNA was diluted to 50 μg μl^−1^ using RNase-free water and 3 μl was used for miRNA profiling on the multiplexed nCounter Human v2 miRNA platform (NanoString Technologies, Seattle, WA, USA)^53^. RNA samples were prepared by ligating a specific DNA tag (miR-tag) onto the 3′ end of each mature miRNA as per the manufacturer's instructions. Excess tags were removed by restriction digestion at 37 °C. Hybridizations were carried out by combining 5 μl of each miRNA-Tag sample with 20 μl of nCounter Reporter probes in hybridization buffer and 5 μl of nCounter Capture probes at 65 °C for 16–20 h. Excess probes were removed using a two-step magnetic bead-based purification on the nCounter Prep Station. Abundance of specific target molecules was quantified using the nCounter Digital Analyzer by counting the individual fluorescent barcodes and assessing target molecules. The data were collected using the nCounter Digital Analyzer and were analysed using the nCounter Analysis system as per the manufacturer's instruction.

### Microarray and pathway enrichment analyses

Microarray was performed on Human HT-12 v4 Beadchip (Illumina) as per the manufacturer's protocol^50^. The SKOV3ip1 cells were treated with control miRNA or miR-192 (40 nM) using RNAiMAX transfection reagent. Total RNA was extracted 48 h following transfection using mirVana RNA isolation labeling kit (Ambion). RNA purity was assessed by a Nanodrop spectrophotometric measurement (Thermo Scientific) of the OD260/280 ratio with acceptable values falling between 1.9 and 2.1. Five hundred nanograms of total RNA were used for labelling and hybridization according to the manufacturer's protocols. After the bead chips were scanned with an Illumina BeadArray Reader (Illumina, San Diego, CA, USA), the microarray data were normalized using the quantile normalization method in the Linear Models for Microarray Data package in the R language environment. The expression level of each gene was transformed into a log_2_ base before further analysis. Ingenuity IPA software was used to identify the genes that are most significantly downregulated following the miR-192 treatment. The pathway enrichment analyses were performed using NetWalker software^16^,^54^. Briefly, scoring of network components (nodes, edges) for their relevance to a given data set is done by a random walk-based scoring strategy by using the data values of nodes as transition probabilities. Each interaction is assigned a probability value based on the data values of nodes in the neighbourhood. Final relevance scores of nodes are given by their visitation frequencies by the random walk at the end of infinite iterations.

### Immunohistochemistry and immunofluorescence analyses

Staining was performed in paraffin-embedded tumour sections as described previously^16^,^49^. For patient samples, tissue microarray slides containing 128 ovarian cancer samples were obtained from Wayne State University following approval by the Institutional Review Board. All participants provided written informed consent. The three most hypervascular areas in each tumour sample were selected for MVD assessment (CD34 staining, 1:20, BioGenex Laboratories). The mean value for the three fields was recorded and was expressed as the number of vessels per HPF ( × 200). For EGR1 and HOXB9 staining, antigen retrieval was performed using Borg Decloaker solution (Biocare Medical). The slides were subsequently incubated with primary antibodies against EGR1 (1:50, Cell Signaling, Catalogue number 4153) or HOXB9 (1:200, Abcam, Catalogue number ab66765) at 4 °C overnight, followed by incubation with DAKO EnVision Dual link system-HRP (Dako). The stained slides were scored by two investigators blinded to patient survival information or miR-192 expression levels. A scoring system, which considered both the intensity of staining and the percentage of cells stained, was used^55^,^56^. H scores of >100 and ≤100 were defined as high and low expression, respectively. All the slides were counterstained with Gill's hematoloxylin #3.

For cleaved caspase-3 staining, formalin-fixed paraffin sections were deparaffinized, and endogenous peroxide was blocked by incubating the slides with 3% hydrogen peroxide in PBS. The slides were incubated in 4% fish gelatin in PBS, followed by an overnight incubation with the cleaved caspase-3 primary antibody (1:100, Biocare Medical, Catalogue number CP229C). The slides were then incubated with biotinylated goat anti-rabbit secondary antibody (Biocare Medical, Catalogue number GR602H) for 20 min, after which they were washed with PBS and were later incubated with streptavidin HRP label (Biocare Medical, Catalogue number HP604H) for 20 min. All the slides were counterstained with Gill's hematoloxylin #3.

For CD31 and desmin staining, optimal cutting temperature embedded frozen tissue sections of human or mouse tumours were used. The slides were incubated with primary antibody for human CD31 (1:25, Dako, Catalogue number M0823), mouse CD-31 (1:800, BD Pharmingen, Catalogue number 553370) and mouse desmin (1:200, Abcam, Catalogue number ab15200) overnight at 4 °C. For human samples, two most hypervascular areas in each tumour sample were selected for MVD assessment. The mean value for the two fields was recorded and was expressed as the number of vessels per HPF ( × 200). For *in vivo* samples, 5–10 random fields at × 200 magnification for each tumour were examined. A vessel was defined as an open lumen with at least one adjacent CD31-positive cell. Multiple positive cells beside a single lumen were counted as one vessel. For pericyte coverage analysis, five random fields at × 200 magnification were taken per slide (five tumours per group), and the percentage of CD-31 staining blood vessels with at least 50% pericyte coverage (positive desmin staining) were enumerated per HPF. The quantification of extravascular FITC-dextran was performed using the following scoring system^16^: 0 points, no staining; 1 point, focal or <25%; 2 points, 25–50%; 3 points, 50–75%; and 4 points, 75–100% FITC-dextran (use three fields per tumour at × 400 magnification). For all the immunofluorescent analyses, nuclear staining was performed with Hoechst 33342 (1:10,000, Molecular Probes).

### MiRNA *in situ* hybridization

Tisue microarray samples for ovarian tumours were used for miR-192 quantification. The formalin-fixed paraffin-embedded tissue sections were dewaxed in xylenes, and rehydrated through an ethanol dilution series. The tissue sections were digested with 15 μg ml^−1^ proteinase K for 20 min at room temperature, then loaded onto Ventana Discovery Ultra for *in situ* hybridization analysis^16^. The tissue slides were incubated with double-DIG-labelled mercury LNA miR-192 probe (Exiqon) for 2 h at 52 °C. The double-DIG-labelled control U6 snRNA probe (Exiqon) was used as a positive control. The digoxigenins were then detected with a polyclonal anti-DIG antibody and alkaline phosphatase conjugated second antibody (Ventana) using NBT-BCIP as the substrate. Representative light field images were obtained using a Nikon Microphot-FXA microscope and Leica DFC320 digital camera. The expression levels of miR-192 were determined using CellProfiler 2.0 software to establish the staining intensity threshold levels and to quantify the number of positively stained cancer cells per HPF ( × 200 magnification)^16^.

### Transcription factor analysis and target gene binding site prediction

Matbase (Genomatix Inc.) was used to identify potential transcription factor-gene/miRNA interactions. A matrix similarity score cutoff of 0.9 or 0.95 were used for identifying transcription factors that can bind directly to the promoter regions of the pro-angiogenic genes or miR-192, respectively. MiRwalk database^57^ was subsequently used to identify transcription factors that can be directly targeted by miR-192. The transcription factor–gene interaction was then plotted using Cytoscape 3.2.1 software.

The putative miR-192 binding sites for a panel of transcription factors, including *EGR1* and *HOXB9*, were assessed bioinformatically using several algorithms^16^. This was performed utilizing the following publically available sites: http://www.microrna.org for the miRanda algorithm, http://www.targetscan.org for the TargetScan algorithm, http://genie.weizmann.ac.il/pubs/mir07 for the PITA algorithm, http://cbcsrv.watson.ibm.com for the RNA22 algorithm, http://diana.cslab.ece.ntua.gr/microT for microT algorithm and http://genome.ucsc.edu/cgi-bin/hgTables?command=start together with http://pictar.mdc-berlin.de/ for the PicTar algorithm. We used Perl to retrieve and sort the information available through these sources and Latex to present the miRNA binding sites most probable to interact.

### Generation of miR-192/EGR1/HOXB9 expressing cell lines

Stable lentiviral miRNA vectors were purchased from Thermo Scientific (HMR5887 for non-targeting control and V1SMHS_000952 for miR-192). The SKOV3ip1 cells were transfected with each lentiviral vector and were selected with growth medium containing puromycin according to the manufacturer's protocol. To generate *EGR1* and/or *HOXB9* expressing cell lines, lentiviral vectors were generated from *EGR1* (GE Healthcare) and *HOXB9* vectors (Origene) lacking 3′-UTR region. Following viral transduction, the cells were selected or sorted using puromycin or GFP for *EGR1* and *HOXB9*, respectively.

### Tube formation assays

The RF24 cells (2 × 10^4^) were plated into each well in Matrigel-coated 96-well plates (50 μl per well, BD Biosciences)^16^. The cells were incubated with conditioned media collected from stable SKOV3ip1 control miRNA or miR-192 expressing cells or from ovarian or renal cancer cells treated control miRNA, miR-192, miR-194 or miR-215 mimics. For the rescue experiments, IL-6 (8,000 pg ml^−1^, R&D systems) and IL-8 (13,000 pg ml^−1^, R&D systems) cytokines were added to the conditioned media. Five wells were used per treatment group and following 6 h of incubation, the images were taken using an Olympus IX81 inverted microscope ( × 100 or × 200 magnification). The number of nodes (defined as when at least three cells formed a single point) per image was quantified.

### Endothelial cell extraction

After approval by the Institutional Review Board at M.D. Anderson Cancer Center, three high-grade serous ovarian carcinomas and three normal ovarian tissue samples were obtained immediately following the resection. The endothelial cells were then extracted from tissues^16^. The samples were treated with 5 mM DTT and 10 mM EDTA before being transferred to a small dish where a few drops of digestion cocktail (Elastase 250 μg ml^−1^, collagenase A 2 mg ml^−1^, DNAse 10 u ml^−1^) were added. The tissues were then minced and were further digested with collagenase A (1 gram tissue in 10 ml) at 37 °C for 1 h. The cells were then spun down at 1,000 r.p.m. for 5 min at room temperature and were re-suspended in 10 ml culture media (MEM medium, 15% FBS, 0.5% gentomycin, 10 ng ml^−1^ bFGF, 2 mM L-glutamine, 1 mM Na Pyruvate, 1 × MEM NEAA and vitamins). The cells were then loaded into Ficoll tubes and were pelleted at 1,800 r.p.m. at room temperature for 20 min. The 2–5 ml interface layered cells were collected, counted and re-suspended in a PBS/BSA buffer solution at 10^7^ cells ml^−1^. The epithelial cells were removed by BerEP4 magnetic beads using a magnet. Finally, a double-positive selection for endothelial cells was performed using anti-CD31 and anti-CD146 antibodies by FACS sorting.

### Statistical analysis

Statistical analysis for tumoral miR-192, EGR1 and HOXB9 expression in patients was performed as previously described^55^,^56^. Kaplan–Meier survival curves were generated and compared with the use of a log-rank statistic to assess the effect of tumoral miR-192, EGR1 or HOXB9 expression on overall survival. The Spearman test was used to assess the correlation between miR-192 and gene expression or angiogenic scores. For other assays, Student's *t*-test was performed to examine the difference between the control and treatment groups. A *P* value less than 0.05 was deemed statistically significant. All the statistical tests were two-sided.

## Additional information

**Accession codes:** The microarray data have been deposited in the GEO database under accession code GSE69990.

**How to cite this article:** Wu, S. Y. *et al*. A miR-192-EGR1-HOXB9 regulatory network controls the angiogenic switch in cancer. *Nat. Commun.* 7:11169 doi: 10.1038/ncomms11169 (2016).

## Supplementary Material

Supplementary Information Supplementary Figures 1-7 and Supplementary Tables 1-8.

Supplementary Data 1 Nanostring miRNA analyses of poorly and highly angiogenic human HGSC tumors

Supplementary Data 2 Median expression of each angiogenic factor in the TCGA ovarian cancer dataset.

Supplementary Data 3 Genomic analyses of SKOV3ip1 cells treated with control miRNA and miR-192.

Supplementary Data 4 Functional pathways altered by miR-192.
