# Supplementary material for: A miR-192-EGR1-HOXB9 regulatory network controls the angiogenic switch in cancer
Source: Nat Commun. 2016 Apr 4;7:11169. doi: 10.1038/ncomms11169 (PMC4822037; doi:10.1038/ncomms11169)
Supplement: Supplementary Information — Supplementary Figures 1-7 and Supplementary Tables 1-8. [file ncomms11169-s1.pdf]

Supplementary Figures

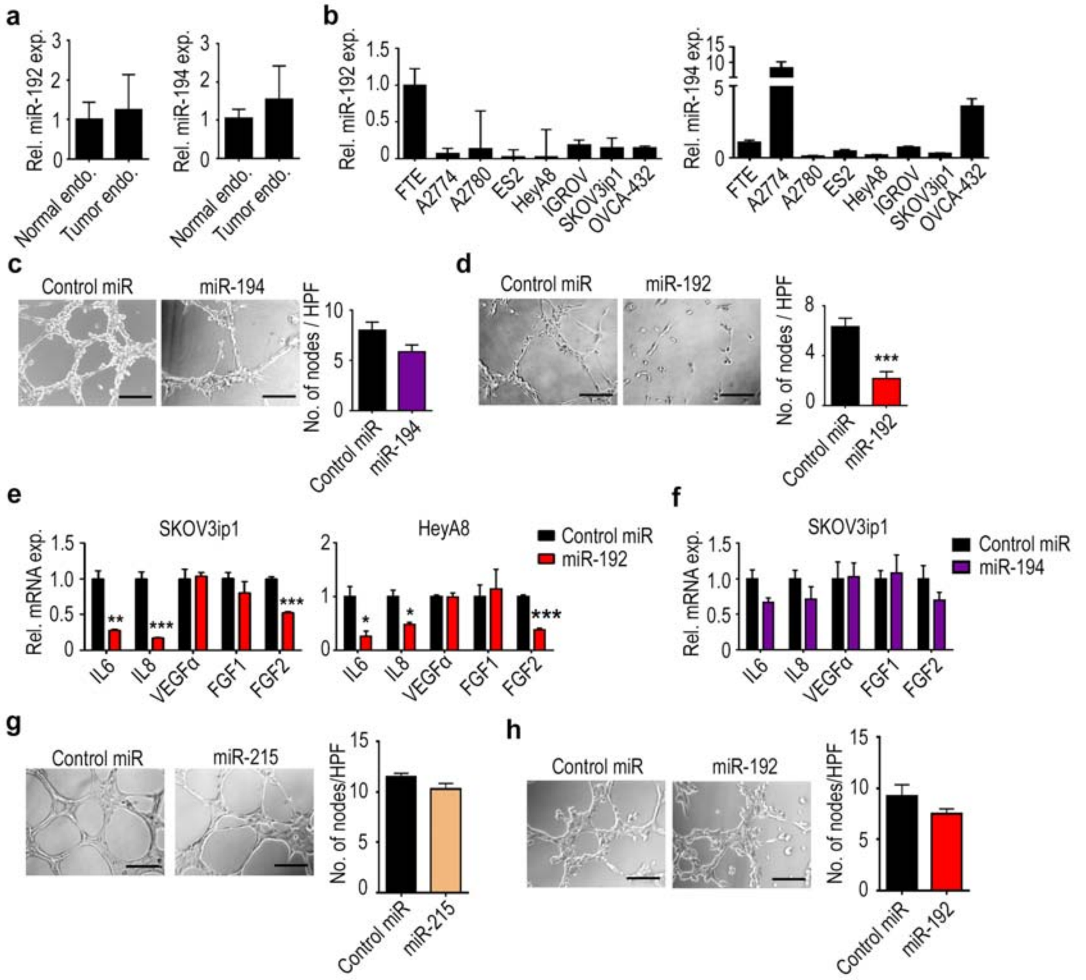

Supplementary Figure 1. The effects of miR-192 and miR-194 on angiogenesis. (**a**) The expression of miR-192 and miR-194 in endothelial cells isolated from normal ovary (n=3) or ovarian tumors (n=3). (**b**) Expression of miR-192 and miR-194 in a panel of ovarian cancer cells. Fallopian tube epithelial (FTE) cells were used as a control. (**c**) Tube formation potential of RF24 cells was assessed after exposure to conditioned media collected from SKOV3ip1 cells treated with control miRNA or miR-194 (48 hrs post transfection). Tube formation was assessed 6 hrs post incubation (n=5). (**d**) The effect of miR-192 on tube formation potential. RF24 cells were exposed to conditioned media collected from HeyA8 cells treated with control miRNA or miR-192 (48 hrs post transfection, n=5, student *t*-test). (**e**) mRNA levels of several important angiogenic factors following control miRNA or miR-192 transfection in SKOV3ip1 or HeyA8 cells (n=3, student *t*-test). (**f**) mRNA levels of angiogenic factors following control miRNA or miR-194 transfection in SKOV3ip1 cells (n=3). (**g**) The effect of miR-215 on tube formation potential. RF24 cells were exposed to conditioned media collected from SKOV3ip1 cells treated with control miRNA or miR-215 (n=5). (**h**) Tube formation potential of RF24-Control miR and RF24-miR-192 stable expressing cells (n=5). Scale bar, 100 µm. All bars and error bars represent mean values and the corresponding SEMs. (*, $P<0.05$; **, $P<0.01$; and ***, $P<0.001$).

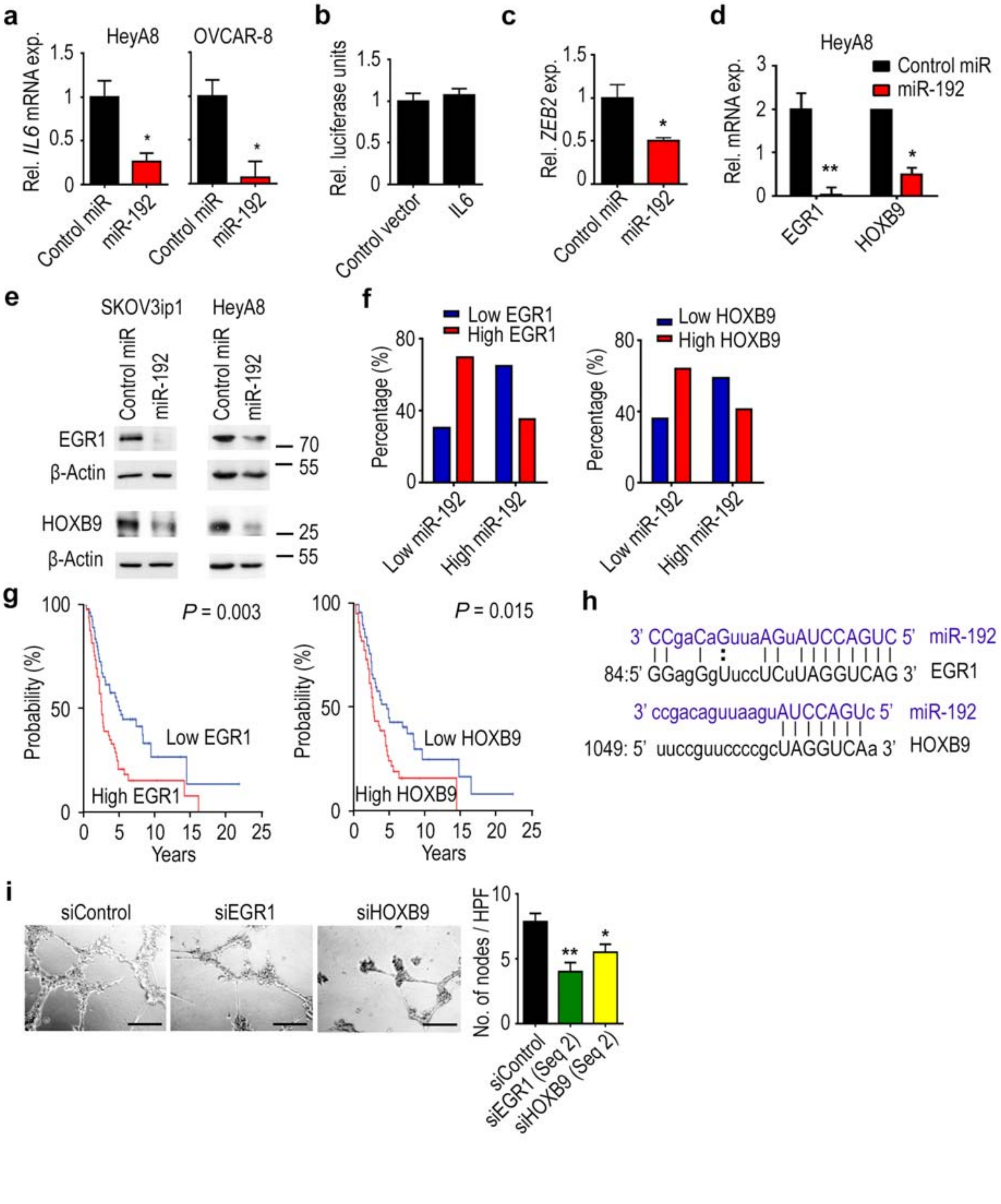
a
HeyA8
OVCAR-8
Rel. IL6 mRNA exp.
Control miR
miR-192
b
Rel. luciferase units
Control vector
IL6
c
Rel. ZEB2 exp.
Control miR
miR-192
d
HeyA8
Control miR
miR-192
Rel. mRNA exp.
EGR1
HOXB9
e
SKOV3ip1
HeyA8
Control miR
miR-192
EGR1
β-Actin
HOXB9
β-Actin
70
55
25
55
f
Low EGR1
High EGR1
Low HOXB9
High HOXB9
Percentage (%)
Low miR-192
High miR-192
g
P = 0.003
P = 0.015
Probability (%)
Low EGR1
High EGR1
Low HOXB9
High HOXB9
Years
h
3' CCgaCaGuuaAGuAUCCAGUC 5' miR-192
84:5' GGagGgUuccUCuUAGGUCAG 3' EGR1
3' ccgacaguuaaguAUCCAGUc 5' miR-192
1049: 5' uuccguuccccgcUAGGUCAa 3' HOXB9
i
siControl
siEGR1
siHOXB9
No. of nodes / HPF
siControl
siEGR1 (Seq 2)
siHOXB9 (Seq 2)

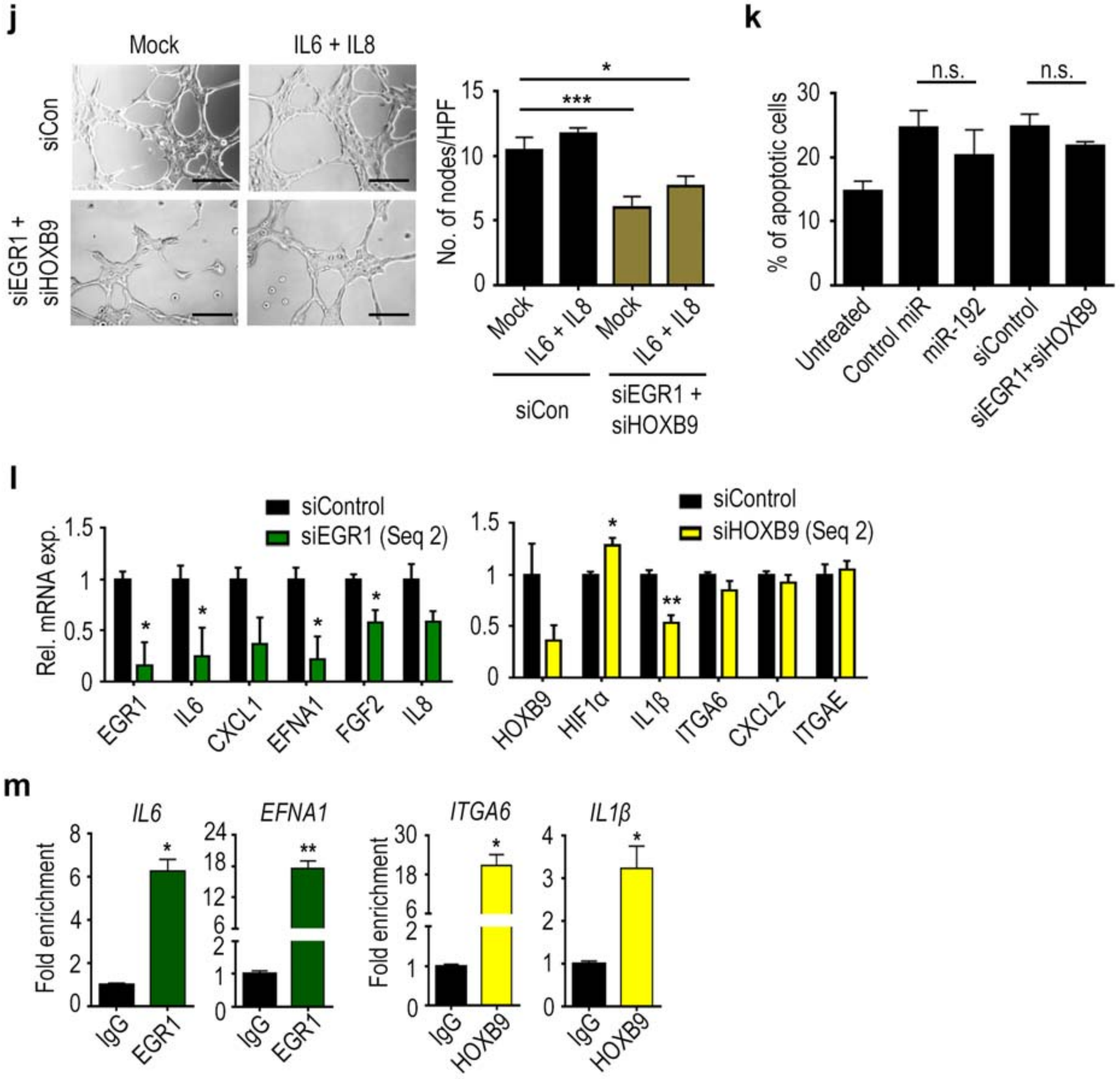

Supplementary Figure 2. The mechanism by which miR-192 mediates its broad anti-angiogenic function. (**a**) The effect of miR-192 on *IL6* level in HeyA8 and OVCAR-8 cells. mRNA levels were assessed at 48 hrs post-transfection (n=3, student *t*-test). (**b**) Relative luciferase activity normalized to empty control for *IL6* 3'-UTR. SKOV3ip1 cells were transfected with miR-192 and UTR luciferase constructs and luciferase assay was performed at 24 hrs post transfection (n=5, student *t*-test). (**c**) Effect of miR-192 treatment on *ZEB2* expression in SKOV3ip1 cells. mRNA levels were assessed at 48 hrs post transfection (n=3, student *t*-test). (**d**) Effect of miR-192 on EGR1 and HOXB9 mRNA levels in HeyA8 cells at 24 hrs post-transfection (n=3, student *t*-test). (**e**) Effect of miR-192 on EGR1 and HOXB9 protein levels at 48 hrs post-transfection in SKOV3ip1 and HeyA8 cells. (**f**) Correlation between miR-192 expression and EGR1 and HOXB9 protein levels in human ovarian epithelial tumors. Tumors were dichotomized into high vs. low

EGR1 or HOXB9 expression groups and the percentages of patients in each of these groups were assessed in tumors with high *vs.* low miR-192 expression (n=98, Chi-square test). (**g**) The impact of tumoral EGR1 or HOXB9 protein expression on overall survival in ovarian cancer patients (n=98, log-rank test). (**h**) Predicted miR-192 binding sites for *EGR1* and *HOXB9*. (**i**) Tube formation potential was assessed in RF24 cells after exposing to conditioned media collected from HeyA8 cells treated with siEGR1 or siHOXB9 (48 hrs post-transfection, n=5, student *t*-test). (**j**) Tube formation assay in RF24 cells following incubation with conditioned media collected from SKOV3ip1 cells treated with control siRNA or siEGR1+siHOXB9 (48 hrs post- transfection). The assay was performed in the presence or absence of IL-6 (8,000 pg/mL) and IL-8 (13,000 pg/mL). Bar graph shows the quantitative analyses of the number of nodes per HPF (n=5, student *t*-test). (**k**) The effect of miR-192 and siEGR1+siHOXB9 treatments on the survival of RF24 cells. RF24 cells were exposed to conditioned media collected from SKOV3ip1 cells treated with miR-192 or siEGR1+siHOXB9 (48 hrs post- treatment). After 6 hrs of exposure, the number of apoptotic cells was assessed *via* Annexin V and propidium iodide staining (n=3, student *t*-test). (**l**) Effect of EGR1 or HOXB9 silencing on levels of angiogenic factors in HeyA8 cells. RNA was isolated at 48 hours post transfection (n=3, student *t*-test). (**m**) Binding of EGR1 or HOXB9 to the promoter regions of *IL6*, *EFNA1*, *ITGA6*, or *IL1β* in SKOV3ip1 cells (n=2, student *t*-test). Scale bar, 100 μm. All bars and error bars represent mean values and the corresponding SEMs (*, $P<0.05$; **, $P<0.01$; and ***, $P<0.001$).

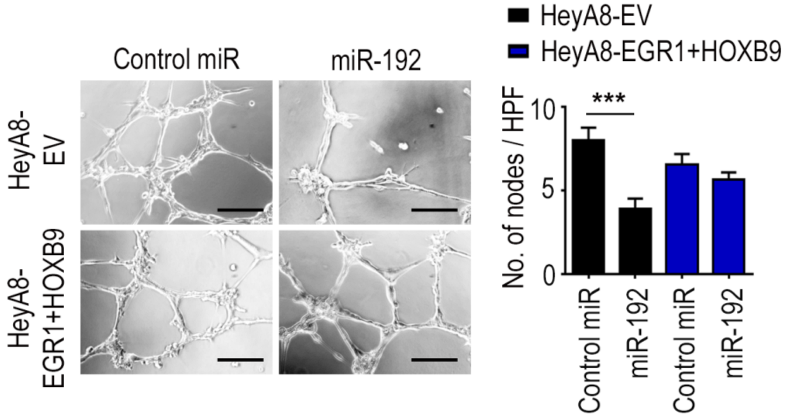

Supplementary Figure 3. The role of EGR1 and HOXB9 in mediating the anti-angiogenic effect of miR-192. RF24 cells were incubated with conditioned media collected from control miRNA or miR-192 treated HeyA8-EV or HeyA8-EGR1+HOXB9 cells (48 hrs post-transfection). Tube formation potential was assessed at 6 hrs post-incubation (n=5, student *t*-test). Scale bar, 100 µm. Bars and error bars represent mean values and the corresponding SEMs (***, $P<0.001$).

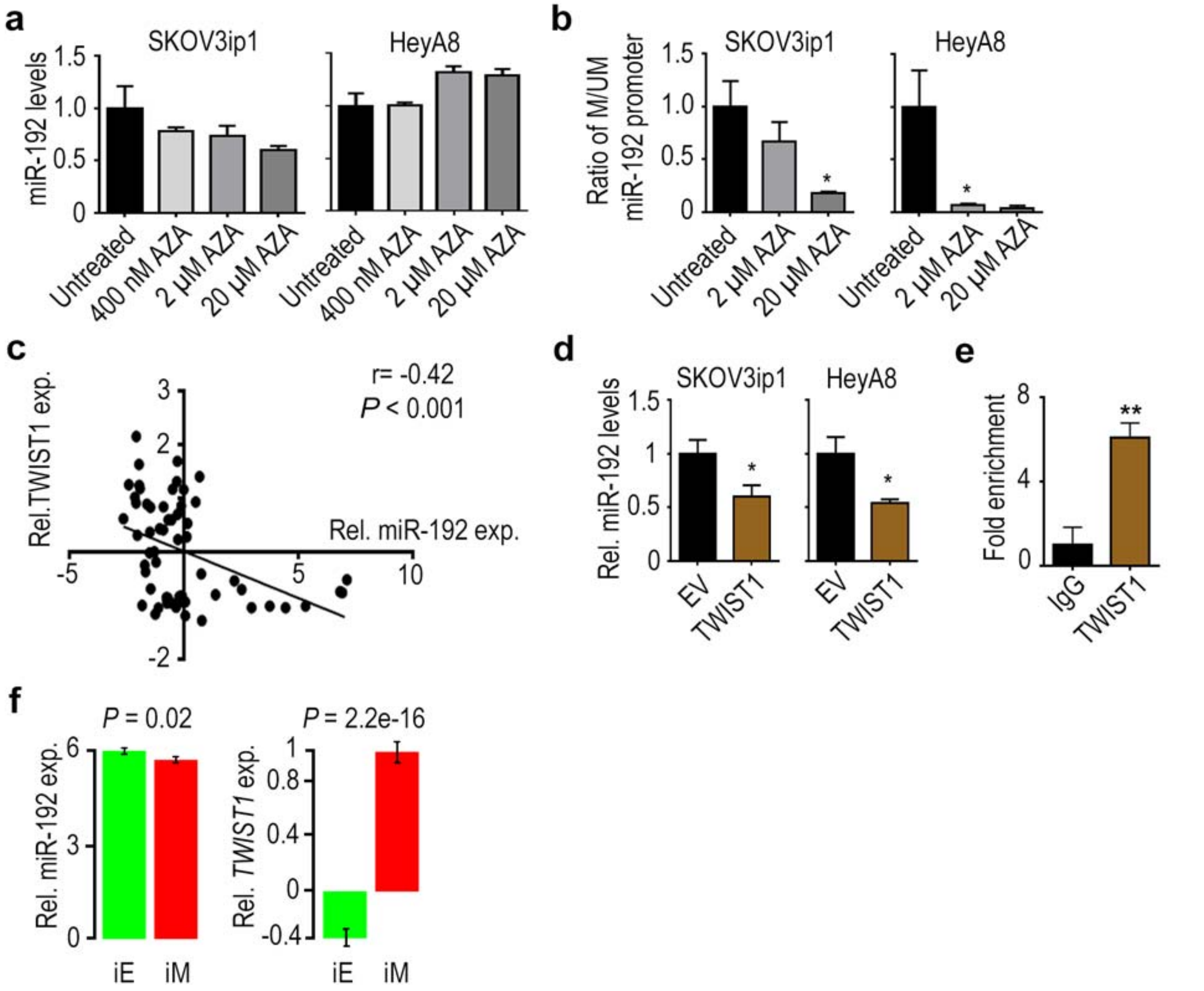

Supplementary Figure 4. Upstream regulation of miR-192. (**a**) MiR-192 expression level at 72 hrs following azacitidine (AZA) treatment in SKOV3ip1 and HeyA8 cells. (**b**) The effect of azacitidine on the ratios of methylated (M) / unmethylated (UM) miR-192 promoter levels (n=3, student *t*-test). (**c**) Correlation between miR-192 and *TWIST1* expression in NCI-60 cell line database (*P*<0.001, Spearman correlation test). (**d**) Effect of TWIST1 expression on miR-192 levels in SKOV3ip1 and HeyA8 cells. Empty vector transfected cells were used as the control (n=3, student *t*-test). (**e**) Binding of TWIST1 to the promoter regions of miR-192 in OVCA-432 high-grade serous ovarian cancer cells (n=2, student *t*-test). (**f**) Relative TWIST1 and miR-192 expression in integrative epithelial (iE) *vs.* integrative mesenchymal (iM) subtype of ovarian tumors (n=459, Wilcoxon rank-sum test). All bars and error bars represent mean values and the corresponding SEMs (*, *P*<0.05; **, *P*<0.01).

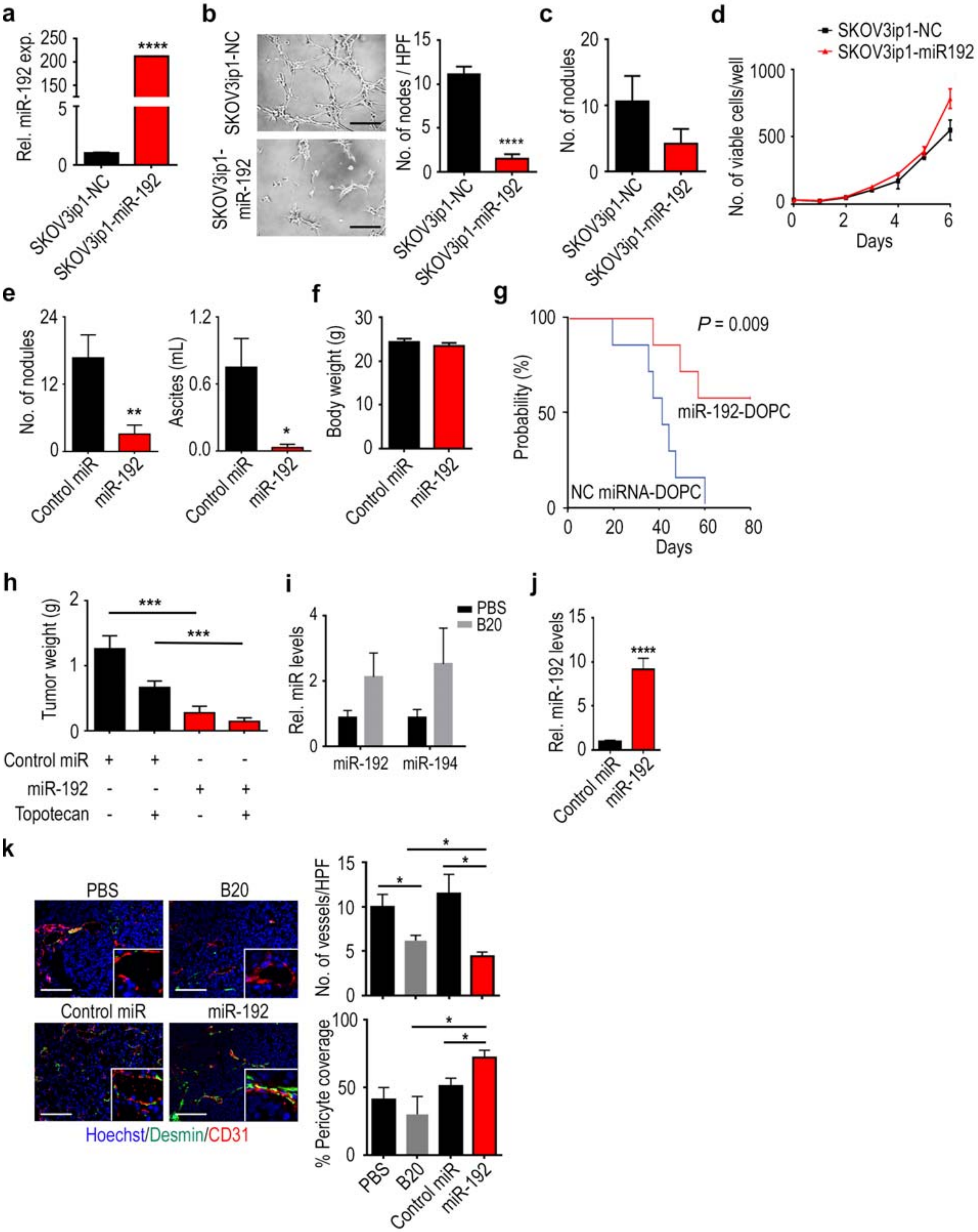

a
Rel. miR-192 exp.
****
SKOV3ip1-NC
SKOV3ip1-miR-192
b
SKOV3ip1-NC
SKOV3ip1-miR-192
No. of nodes / HPF
****
SKOV3ip1-NC
SKOV3ip1-miR-192
c
No. of nodules
SKOV3ip1-NC
SKOV3ip1-miR-192
d
SKOV3ip1-NC
SKOV3ip1-miR192
No. of viable cells/well
Days
e
No. of nodules
**
Ascites (mL)
*
Control miR
miR-192
f
Body weight (g)
Control miR
miR-192
g
P = 0.009
Probability (%)
miR-192-DOPC
NC miRNA-DOPC
Days
h
Tumor weight (g)
***
***
Control miR + + - -
miR-192 - - + +
Topotecan - + - +
i
PBS
B20
Rel. miR levels
miR-192
miR-194
j
Rel. miR-192 levels
****
Control miR
miR-192
k
PBS
B20
Control miR
miR-192
Hoechst/Desmin/CD31
No. of vessels/HPF
% Pericyte coverage
PBS
B20
Control miR
miR-192

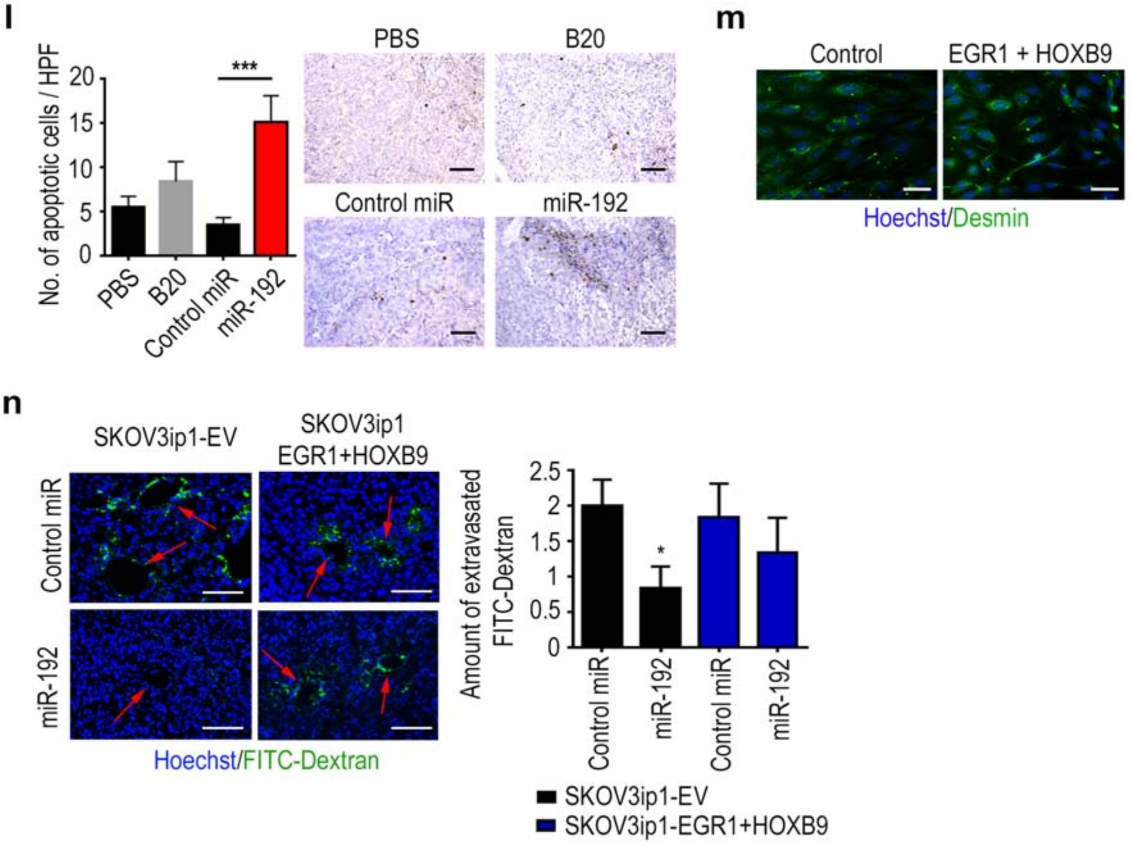

Supplementary Figure 5. *In vivo* effects of miR-192 in mouse models of ovarian cancer. (**a**) Expression of miR-192 in SKOV3ip1-NC and SKOV3ip1-miR-192 cells (n=3, student *t*-test). (**b**) Tube formation potential of RF24 cells treated with conditioned media collected from SKOV3ip1-NC and SKOV3ip1-miR-192 cells (n=5, student *t*-test). Both cell lines were seeded at 300,000 cells per well and conditioned media was collected at 48 hrs post seeding. Scale bar, 100 µm. (**c**) Number of tumor nodules in mice bearing SKOV3ip1-NC or SKOV3ip1-miR-192 tumors. (**d**) The effect of miR-192 expression on growth of SKOV3ip1 cells *in vitro* (n=3). (**e**) The effect of miR-192-DOPC therapy on the number of tumor nodules and volume of ascites in SKOV3ip1 tumor bearing mice (n=10, student *t*-test). (**f**) The effects of miR-192-DOPC therapy on mouse body weight (n=10). (**g**) The impact of miR-192-DOPC treatment on survival in mice. Mice bearing SKOV3ip1 tumors were treated with control miRNA-DOPC or miR-192-DOPC twice weekly. A Kaplan Meier curve was used to analyze the survival difference between control and treatment groups (n=7, log-rank test). (**h**) The effect of miR-192-DOPC on tumor burden in mice bearing SKOV3ip1 tumors treated with control miRNA-DOPC, miR-192-DOPC, and/or topotecan (n=10, student *t*-test). The effect of (**i**) B20 or (**j**) miR-192-DOPC treatment on tumoral miR-192 and/or miR-194 expression (n=5, student *t*-test). The impact of B20 and miR-192-DOPC treatment on (**k**) MVD and vessel pericyte coverage and (**l**) tumor cell apoptosis (n=5, student *t*-test). (**m**) The effect of EGR1 and HOXB9 expression on desmin levels in pericyte-like 10T1/2 cells. 10T1/2 cells were exposed to conditioned media collected from control or EGR1+HOXB9 expressing SKOV3ip1 cells for 48 hrs and desmin expression was assessed *via* immunofluorescence. (**n**)

Representative images of Hoechst 33342 (blue) and FITC-dextran (green) in SKOV3ip1-EV and EGR1+HOXB9 tumors (n=3, student *t*-test). 0 points, no staining; 1 point, focal or <25%; 2 points, 25–50%, 3 points, 50-75%, and 4 points, 75-100% FITC-dextran. Red arrows indicate the location of blood vessels. Scale bar, 50 μm. All bars and error bars represent mean values and the corresponding SEMs (*, $P<0.05$; **, $P<0.01$; ***, $P<0.001$; and ****, $P<0.0001$).

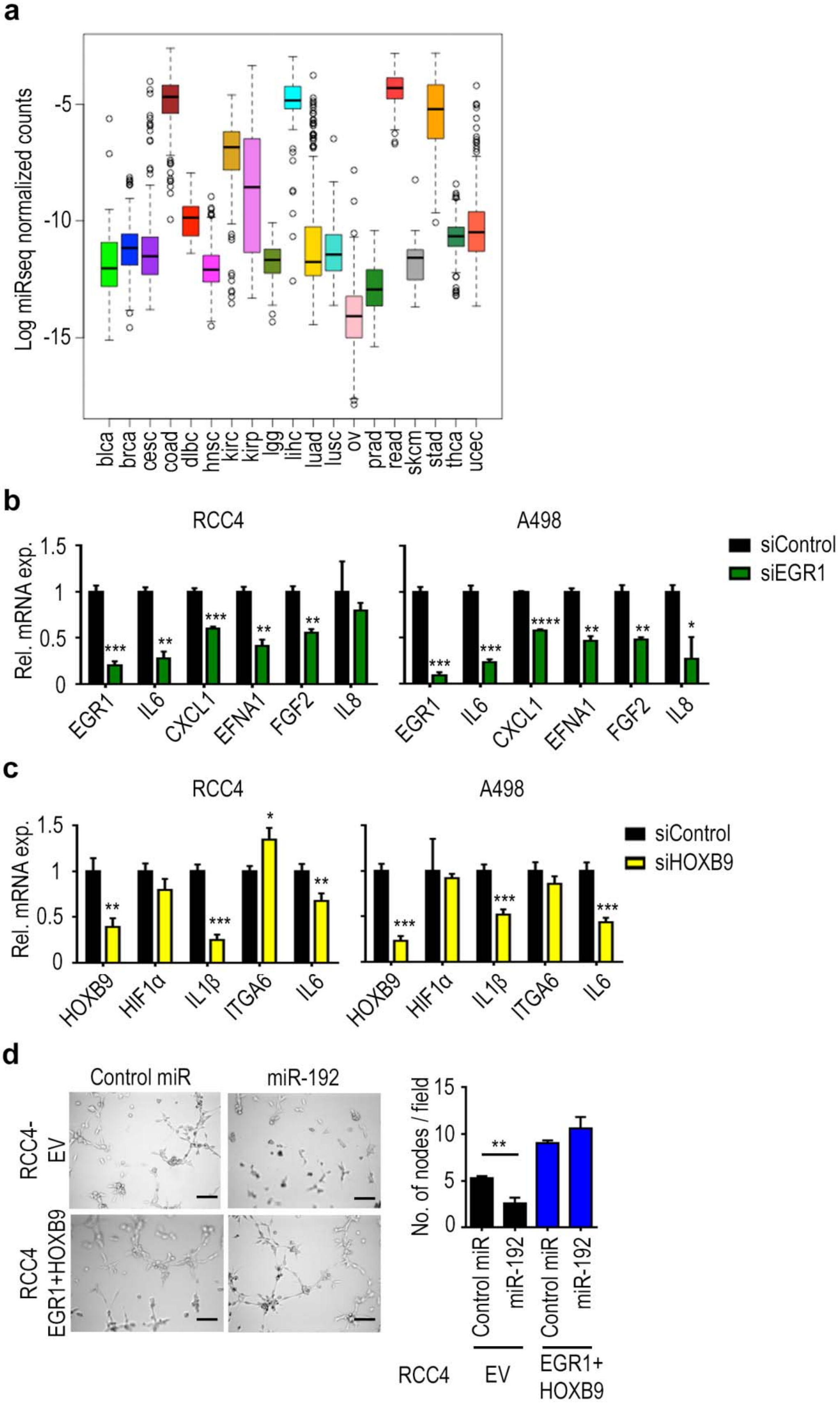
a
Log miRseq normalized counts
-5
-10
-15
blca
brca
cesc
coad
dlbc
hnsc
kirc
kirp
lgg
lihc
luad
lusc
ov
prad
read
skcm
stad
thca
ucec
b
RCC4
A498
siControl
siEGR1
Rel. mRNA exp.
1.5
1
0.5
0
EGR1
IL6
CXCL1
EFNA1
FGF2
IL8
c
RCC4
A498
siControl
siHOXB9
Rel. mRNA exp.
HOXB9
HIF1α
IL1β
ITGA6
IL6
d
Control miR
miR-192
RCC4-EV
RCC4 EGR1+HOXB9
No. of nodes / field
15
10
5
0
Control miR
miR-192
RCC4
EV
EGR1+ HOXB9

Supplementary Figure 6. The expression and functional effect of miR-192 in renal tumors. (**a**) The expression of miR-192 in tumors across 19 cancer types in TCGA databases. The horizontal lines represent the median normalized expression levels. The bottom and the top lines in each individual box represent the first and the third quartile expression levels, respectively. Bladder urothelial carcinoma, BLCA; Breast invasive carcinoma, BRCA; Cervical squamous cell carcinoma and endocervical adenocarcinoma, CESC; Colon adenocarcinoma, COAD; Lymphoid neoplasm diffuse large B-cell lymphoma, DLBC; Head and neck squamous cell carcinoma, HNSC; Kidney renal clear cell carcinoma, KIRC; Kidney renal papillary cell carcinoma, KIRP; Brain lower grade glioma, LGG; Liver hepatocellular carcinoma, LIHC; Lung adenocarcinoma, LUAD; Lung squamous cell carcinoma, LUSC; Ovarian serous cystadenocarcinoma, OV; Prostate adenocarcinoma, PRAD; Rectum adenocarcinoma, READ; Skin Cutaneous Melanoma, SKCM; Stomach adenocarcinoma, STAD; Thyroid carcinoma, THCA; Uterine corpus endometrial carcinoma, UCEC. The levels of angiogenic factors at 48 hrs following (**b**) siEGR1 and (**c**) siHOXB9 treatment in RCC4 and A498 cells (n=3, student *t*-test). (**d**) Tube formation potential of RF24 cells following incubation with conditioned media collected from control miRNA or miR-192 treated RCC4-EV or RCC4-EGR1+HOXB9 cells. Bar graph (right) shows the quantitative analyses of number of nodes per field (n=5, student *t*-test). Scale bar, 100 μm. All bars and error bars represent mean values and the corresponding SEMs (*, $P<0.05$; **, $P<0.01$; ***, $P<0.001$; and ****, $P<0.0001$).

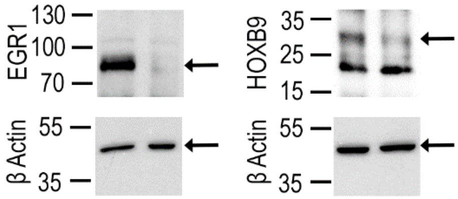

Supplementary Fig. 7. Full scans of Western blots. Full scans of blots from Supplementary Figure 2e.

Supplementary Tables

Supplementary Table 1. Correlation between miRNA levels and angiogenic scores in HGSC tumors (Spearman correlation tests).

	Correlation coefficient (with angiogenic scores)	*P*-values
**miR-96**	-0.2	<0.0001
**miR-181c**	-0.07	0.0873
**miR-182**	-0.13	0.0018
**miR-191**	-0.09	0.0319
**miR-192**	-0.24	<0.0001
**miR-194**	-0.17	<0.0001
**miR-200b**	-0.18	<0.0001
**miR-205**	0.01	0.7908
**miR-522**	0.02	0.6511
**miR-663**	0.02	0.7103

Supplementary Table 2. Correlation of clinical and pathological features with miR-192 levels in 128 patients with invasive epithelial ovarian carcinoma.

Variable		miR-192 Expression		*P* value	Statistical Tests
		Low (N=40)	High (N=88)		
**Tumor grade – no. (%)**	Low<br>High	2<br>38	6<br>82	0.69	Chi-square test
**Tumor stage – no. (%)**	I or II<br>III or IV	5<br>35	14<br>74	0.62	Chi-square test
**Histology – no. (%)**	Serous<br>Others	36<br>4	78<br>10	0.82	Chi-square test
**Cytoreduction – no. (%)**	Optimal<br>Suboptimal	19<br>21	43<br>45	0.89	Chi-square test
**Median OS (Yrs.)**		3.86	6.85	0.001	Log-rank test
**Microvessel density**		106.1	34.6	<0.0001	Student *t*-test

Supplementary Table 3. Top pathways affected by miR-192. Pathway analysis of genes that are negatively associated with miR-192 expression in TCGA ovarian dataset (*P* values were generated using the Fisher Exact Test).

Pathway	No. of molecules	*P* values
Integrin signaling pathway	49	<0.001
Angiogenesis	38	0.004
Notch signaling pathway	12	0.017
Alzheimer disease-presenilin pathway	22	0.048
Wnt signaling pathway	46	0.067
Cadherin signaling pathway	24	0.085

Supplementary Table 4. Transcription factors predicted to be targeted by miR-192.

Transcription Factor	Number of predicted angiogenic factors
ZNF263	18
ZBTB7	17
PLAG1	16
STAT3	14
WT1	14
EGR1	11
TAL1_E2A	11
E2F3	8
GLIS2	8
TEAD	7
HOXB9	6
ZNF217	5
CRX	5

Supplementary Table 5. Correlations between promoter methylation and miR-192 expression levels in TCGA databases. Spearman correlation analysis was used to generate *P* values.

Cancer Types		Methylation Probe			
		MiR192-cg02258444-11-64658622	MiR192-cg09349409-11-64658765	MiR192-cg18262830-11-64658819	MiR192-cg27083891-11-64658726
**BLCA**	**Correlation**	-0.077	-0.112	-0.164	0.031
	***P* values**	0.131	0.029	0.001	0.546
**BRCA**	**Correlation**	-0.031	0.018	0.231	0.001
	***P* values**	0.432	0.651	<0.0005	0.974
**CESC**	**Correlation**	-0.093	-0.245	-0.236	0.027
	***P* values**	0.135	<0.0005	<0.0005	0.662
**COAD**	**Correlation**	-0.594	-0.563	-0.546	-0.576
	***P* values**	<0.0005	<0.0005	<0.0005	<0.0005
**DLBC**	**Correlation**	0.106	0.012	0.124	-0.051
	***P* values**	0.478	0.934	0.404	0.732
**HNSC**	**Correlation**	0.225	0.236	0.258	0.188
	***P* values**	<0.0005	<0.0005	<0.0005	<0.0005
**KIRC**	**Correlation**	-0.627	-0.613	-0.573	-0.612
	***P* values**	<0.0005	<0.0005	<0.0005	<0.0005
**KIRP**	**Correlation**	-0.84	0.764	-0.702	-0.764
	***P* values**	<0.0005	<0.0005	<0.0005	<0.0005
**LGG**	**Correlation**	0.031	0.039	0.16	0.121
	***P* values**	0.477	0.377	<0.0005	0.005
**LIHC**	**Correlation**	-0.652	-0.621	-0.556	-0.642
	***P* values**	<0.0005	<0.0005	<0.0005	<0.0005
**LUAD**	**Correlation**	-0.39	-0.396	-0.364	-0.369
	***P* values**	<0.0005	<0.0005	<0.0005	<0.0005
**LUSC**	**Correlation**	0.164	0.127	0.021	0.221
	***P* values**	0.003	0.02	0.699	<0.0005
**PRAD**	**Correlation**	-0.084	-0.109	0.143	0.123
	***P* values**	0.073	0.02	0.002	0.009
**READ**	**Correlation**	-0.599	-0.605	-0.57	-0.631
	***P* values**	<0.0005	<0.0005	<0.0005	<0.0005
**SKCM**	**Correlation**	-0.039	0.064	0.056	0.094
	***P* values**	0.418	0.179	0.242	0.049
**STAD**	**Correlation**	-0.63	-0.585	-0.553	-0.635
	***P* values**	<0.0005	<0.0005	<0.0005	<0.0005
**THCA**	**Correlation**	-0.101	-0.013	-0.032	-0.05
	***P* values**	0.016	0.753	0.455	0.232
**UCEC**	**Correlation**	-0.124	-0.144	-0.07	-0.126
	***P* values**	0.009	0.003	0.146	0.008

Bladder urothelial carcinoma, BLCA; Breast invasive carcinoma, BRCA; Cervical squamous cell carcinoma and endocervical adenocarcinoma, CESC; Colon adenocarcinoma, COAD; Lymphoid neoplasm diffuse large B-cell lymphoma, DLBC; Head and neck squamous cell carcinoma, HNSC; Kidney renal clear cell carcinoma, KIRC; Kidney renal papillary cell carcinoma, KIRP; Brain lower grade glioma, LGG; Liver hepatocellular carcinoma, LIHC; Lung adenocarcinoma, LUAD; Lung squamous cell carcinoma, LUSC; Ovarian serous cystadenocarcinoma, OV; Prostate adenocarcinoma, PRAD; Rectum adenocarcinoma, READ; Skin Cutaneous Melanoma, SKCM; Stomach adenocarcinoma, STAD; Thyroid carcinoma, THCA; Uterine corpus endometrial carcinoma, UCEC.

Supplementary Table 6. The list of samples used for miRNA-angiogenic factor correlation analyses.

TCGA-04-1331	TCGA-04-1649	TCGA-09-2054	TCGA-13-0765	TCGA-13-0903
TCGA-04-1332	TCGA-04-1651	TCGA-09-2055	TCGA-13-0766	TCGA-13-0904
TCGA-04-1335	TCGA-04-1652	TCGA-09-2056	TCGA-13-0768	TCGA-13-0905
TCGA-04-1336	TCGA-04-1654	TCGA-10-0925	TCGA-13-0791	TCGA-13-0906
TCGA-04-1337	TCGA-04-1655	TCGA-10-0926	TCGA-13-0792	TCGA-13-0908
TCGA-04-1338	TCGA-09-0364	TCGA-10-0927	TCGA-13-0793	TCGA-13-0910
TCGA-04-1342	TCGA-09-0365	TCGA-10-0928	TCGA-13-0794	TCGA-13-0911
TCGA-04-1343	TCGA-09-0366	TCGA-10-0930	TCGA-13-0795	TCGA-13-0912
TCGA-04-1346	TCGA-09-0367	TCGA-10-0931	TCGA-13-0797	TCGA-13-0913
TCGA-04-1347	TCGA-09-0369	TCGA-10-0933	TCGA-13-0799	TCGA-13-0916
TCGA-04-1348	TCGA-09-1659	TCGA-10-0934	TCGA-13-0800	TCGA-13-0919
TCGA-04-1349	TCGA-09-1661	TCGA-10-0935	TCGA-13-0801	TCGA-13-0920
TCGA-04-1350	TCGA-09-1662	TCGA-10-0936	TCGA-13-0802	TCGA-13-0921
TCGA-04-1351	TCGA-09-1664	TCGA-10-0937	TCGA-13-0803	TCGA-13-0923
TCGA-04-1356	TCGA-09-1665	TCGA-10-0938	TCGA-13-0804	TCGA-13-0924
TCGA-04-1361	TCGA-09-1666	TCGA-13-0714	TCGA-13-0805	TCGA-13-1403
TCGA-04-1362	TCGA-09-1667	TCGA-13-0717	TCGA-13-0807	TCGA-13-1404
TCGA-04-1364	TCGA-09-1668	TCGA-13-0720	TCGA-13-0883	TCGA-13-1405
TCGA-04-1365	TCGA-09-1669	TCGA-13-0723	TCGA-13-0884	TCGA-13-1407
TCGA-04-1367	TCGA-09-1670	TCGA-13-0724	TCGA-13-0885	TCGA-13-1408
TCGA-04-1369	TCGA-09-1672	TCGA-13-0725	TCGA-13-0886	TCGA-13-1409
TCGA-04-1371	TCGA-09-1673	TCGA-13-0726	TCGA-13-0887	TCGA-13-1410
TCGA-04-1514	TCGA-09-1674	TCGA-13-0727	TCGA-13-0888	TCGA-13-1411
TCGA-04-1516	TCGA-09-1675	TCGA-13-0730	TCGA-13-0889	TCGA-13-1412
TCGA-04-1517	TCGA-09-2043	TCGA-13-0751	TCGA-13-0890	TCGA-13-1477
TCGA-04-1525	TCGA-09-2044	TCGA-13-0755	TCGA-13-0891	TCGA-13-1481
TCGA-04-1530	TCGA-09-2045	TCGA-13-0757	TCGA-13-0893	TCGA-13-1482
TCGA-04-1542	TCGA-09-2048	TCGA-13-0758	TCGA-13-0894	TCGA-13-1483
TCGA-04-1638	TCGA-09-2049	TCGA-13-0760	TCGA-13-0897	TCGA-13-1484
TCGA-04-1644	TCGA-09-2050	TCGA-13-0761	TCGA-13-0899	TCGA-13-1485
TCGA-04-1646	TCGA-09-2051	TCGA-13-0762	TCGA-13-0900	TCGA-13-1487
TCGA-04-1648	TCGA-09-2053	TCGA-13-0764	TCGA-13-0901	TCGA-13-1488

TCGA-13-1489	TCGA-23-1029	TCGA-24-1417	TCGA-24-1604	TCGA-24-2297
TCGA-13-1491	TCGA-23-1030	TCGA-24-1418	TCGA-24-1614	TCGA-24-2298
TCGA-13-1492	TCGA-23-1031	TCGA-24-1419	TCGA-24-1616	TCGA-25-1312
TCGA-13-1494	TCGA-23-1032	TCGA-24-1422	TCGA-24-1842	TCGA-25-1313
TCGA-13-1495	TCGA-23-1107	TCGA-24-1423	TCGA-24-1843	TCGA-25-1314
TCGA-13-1496	TCGA-23-1109	TCGA-24-1424	TCGA-24-1844	TCGA-25-1315
TCGA-13-1497	TCGA-23-1110	TCGA-24-1425	TCGA-24-1845	TCGA-25-1316
TCGA-13-1498	TCGA-23-1111	TCGA-24-1426	TCGA-24-1846	TCGA-25-1317
TCGA-13-1499	TCGA-23-1113	TCGA-24-1427	TCGA-24-1847	TCGA-25-1318
TCGA-13-1500	TCGA-23-1114	TCGA-24-1428	TCGA-24-1849	TCGA-25-1319
TCGA-13-1501	TCGA-23-1116	TCGA-24-1430	TCGA-24-1850	TCGA-25-1320
TCGA-13-1504	TCGA-23-1117	TCGA-24-1431	TCGA-24-1852	TCGA-25-1321
TCGA-13-1505	TCGA-23-1118	TCGA-24-1434	TCGA-24-1920	TCGA-25-1322
TCGA-13-1506	TCGA-23-1119	TCGA-24-1435	TCGA-24-1923	TCGA-25-1323
TCGA-13-1507	TCGA-23-1120	TCGA-24-1436	TCGA-24-1924	TCGA-25-1324
TCGA-13-1509	TCGA-23-1121	TCGA-24-1463	TCGA-24-1927	TCGA-25-1325
TCGA-13-1510	TCGA-23-1122	TCGA-24-1464	TCGA-24-1928	TCGA-25-1326
TCGA-13-1511	TCGA-23-1123	TCGA-24-1466	TCGA-24-1930	TCGA-25-1328
TCGA-13-1512	TCGA-23-1124	TCGA-24-1467	TCGA-24-2019	TCGA-25-1329
TCGA-13-1817	TCGA-23-1809	TCGA-24-1469	TCGA-24-2020	TCGA-25-1623
TCGA-13-1819	TCGA-23-2072	TCGA-24-1470	TCGA-24-2023	TCGA-25-1625
TCGA-13-2057	TCGA-23-2077	TCGA-24-1471	TCGA-24-2024	TCGA-25-1626
TCGA-13-2059	TCGA-23-2078	TCGA-24-1474	TCGA-24-2026	TCGA-25-1627
TCGA-13-2060	TCGA-23-2079	TCGA-24-1544	TCGA-24-2027	TCGA-25-1628
TCGA-13-2061	TCGA-23-2081	TCGA-24-1545	TCGA-24-2029	TCGA-25-1630
TCGA-13-2065	TCGA-23-2084	TCGA-24-1546	TCGA-24-2030	TCGA-25-1631
TCGA-13-2066	TCGA-23-2641	TCGA-24-1548	TCGA-24-2033	TCGA-25-1632
TCGA-13-2071	TCGA-23-2643	TCGA-24-1549	TCGA-24-2035	TCGA-25-1633
TCGA-20-0987	TCGA-23-2645	TCGA-24-1550	TCGA-24-2036	TCGA-25-1634
TCGA-20-0990	TCGA-23-2647	TCGA-24-1551	TCGA-24-2038	TCGA-25-1635
TCGA-20-0991	TCGA-23-2649	TCGA-24-1552	TCGA-24-2254	TCGA-25-1870
TCGA-20-1682	TCGA-24-0966	TCGA-24-1553	TCGA-24-2260	TCGA-25-1871
TCGA-20-1683	TCGA-24-0968	TCGA-24-1555	TCGA-24-2261	TCGA-25-1877
TCGA-20-1684	TCGA-24-0970	TCGA-24-1556	TCGA-24-2262	TCGA-25-1878
TCGA-20-1685	TCGA-24-0975	TCGA-24-1557	TCGA-24-2267	TCGA-25-2042
TCGA-20-1686	TCGA-24-0979	TCGA-24-1558	TCGA-24-2271	TCGA-25-2391
TCGA-20-1687	TCGA-24-0980	TCGA-24-1560	TCGA-24-2280	TCGA-25-2392
TCGA-23-1021	TCGA-24-0982	TCGA-24-1562	TCGA-24-2281	TCGA-25-2393
TCGA-23-1022	TCGA-24-1103	TCGA-24-1563	TCGA-24-2288	TCGA-25-2396
TCGA-23-1024	TCGA-24-1104	TCGA-24-1564	TCGA-24-2289	TCGA-25-2397
TCGA-23-1026	TCGA-24-1105	TCGA-24-1565	TCGA-24-2290	TCGA-25-2398
TCGA-23-1027	TCGA-24-1413	TCGA-24-1567	TCGA-24-2293	TCGA-25-2399
TCGA-23-1028	TCGA-24-1416	TCGA-24-1603	TCGA-24-2295	TCGA-25-2400

TCGA-25-2401	TCGA-29-2427	TCGA-36-2530	TCGA-61-1728	TCGA-61-2097
TCGA-25-2404	TCGA-29-2428	TCGA-36-2532	TCGA-61-1730	TCGA-61-2098
TCGA-25-2408	TCGA-29-2429	TCGA-36-2534	TCGA-61-1733	TCGA-61-2101
TCGA-25-2409	TCGA-29-2431	TCGA-36-2537	TCGA-61-1734	TCGA-61-2102
TCGA-29-1688	TCGA-29-2432	TCGA-36-2538	TCGA-61-1736	TCGA-61-2104
TCGA-29-1690	TCGA-29-2434	TCGA-36-2540	TCGA-61-1737	TCGA-61-2109
TCGA-29-1691	TCGA-29-2436	TCGA-36-2542	TCGA-61-1738	TCGA-61-2110
TCGA-29-1692	TCGA-30-1714	TCGA-36-2543	TCGA-61-1740	TCGA-61-2111
TCGA-29-1693	TCGA-30-1718	TCGA-36-2544	TCGA-61-1741	TCGA-61-2113
TCGA-29-1694	TCGA-30-1853	TCGA-36-2545	TCGA-61-1743	TCGA-61-2612
TCGA-29-1695	TCGA-30-1855	TCGA-36-2547	TCGA-61-1895	TCGA-61-2613
TCGA-29-1696	TCGA-30-1856	TCGA-36-2548	TCGA-61-1899	TCGA-61-2614
TCGA-29-1697	TCGA-30-1857	TCGA-36-2549	TCGA-61-1900	
TCGA-29-1698	TCGA-30-1859	TCGA-36-2551	TCGA-61-1901	
TCGA-29-1699	TCGA-30-1860	TCGA-36-2552	TCGA-61-1903	
TCGA-29-1701	TCGA-30-1861	TCGA-42-2582	TCGA-61-1904	
TCGA-29-1702	TCGA-30-1862	TCGA-42-2587	TCGA-61-1906	
TCGA-29-1703	TCGA-30-1866	TCGA-42-2588	TCGA-61-1907	
TCGA-29-1704	TCGA-30-1867	TCGA-42-2589	TCGA-61-1910	
TCGA-29-1705	TCGA-30-1880	TCGA-42-2590	TCGA-61-1913	
TCGA-29-1707	TCGA-30-1887	TCGA-42-2591	TCGA-61-1914	
TCGA-29-1710	TCGA-30-1891	TCGA-57-1582	TCGA-61-1915	
TCGA-29-1711	TCGA-30-1892	TCGA-57-1583	TCGA-61-1916	
TCGA-29-1761	TCGA-31-1944	TCGA-57-1584	TCGA-61-1917	
TCGA-29-1762	TCGA-31-1946	TCGA-57-1585	TCGA-61-1918	
TCGA-29-1763	TCGA-31-1950	TCGA-57-1586	TCGA-61-1919	
TCGA-29-1764	TCGA-31-1951	TCGA-57-1992	TCGA-61-1995	
TCGA-29-1766	TCGA-31-1953	TCGA-57-1993	TCGA-61-1998	
TCGA-29-1768	TCGA-31-1955	TCGA-57-1994	TCGA-61-2000	
TCGA-29-1769	TCGA-31-1956	TCGA-59-2348	TCGA-61-2002	
TCGA-29-1770	TCGA-31-1959	TCGA-59-2349	TCGA-61-2003	
TCGA-29-1771	TCGA-36-1568	TCGA-59-2350	TCGA-61-2008	
TCGA-29-1774	TCGA-36-1569	TCGA-59-2351	TCGA-61-2009	
TCGA-29-1775	TCGA-36-1570	TCGA-59-2352	TCGA-61-2012	
TCGA-29-1776	TCGA-36-1571	TCGA-59-2354	TCGA-61-2016	
TCGA-29-1777	TCGA-36-1574	TCGA-59-2355	TCGA-61-2017	
TCGA-29-1778	TCGA-36-1575	TCGA-59-2363	TCGA-61-2018	
TCGA-29-1781	TCGA-36-1576	TCGA-59-2372	TCGA-61-2087	
TCGA-29-1783	TCGA-36-1577	TCGA-61-1721	TCGA-61-2088	
TCGA-29-1784	TCGA-36-1578	TCGA-61-1722	TCGA-61-2092	
TCGA-29-1785	TCGA-36-1580	TCGA-61-1724	TCGA-61-2094	
TCGA-29-2414	TCGA-36-1581	TCGA-61-1725	TCGA-61-2095	
TCGA-29-2425	TCGA-36-2529	TCGA-61-1727	TCGA-61-2096	

Supplementary Table 7. List of siRNA sequences used in this study

Gene	SiRNA Sequences
siControl	5'-UUAUGCCGAUCGCGUCACA**TT**-3'<br>3'-**TT**AAUACGGCUAGCGCAGUGU-5'
siEGR1 (Seq 1)	5'-CCAACGACAGCAGUCCCAU**TT**-3'<br>3'-**TT**GGUUGCUGUCGUCAGGGUA-5'
siEGR1 (Seq 2)	5'-CAAAGAACUUGAUUUGCAU**TT**-3'<br>3'-**TT**GUUUCUUGAACUAAACGUA-5'
siHOXB9 (Seq 1)	5'-CUAAGAAUGUGAUGGGCCA**TT**-3'<br>3'-**TT**GAUUCUUACACUACCCGGU-5'
siHOXB9 (Seq 2)	5'-CUUGCCUGUUUCUUCUCUA**TT**-3'<br>3'-**TT**GAACGGACAAAGAAGAGAU-5'

Supplementary Table 8. Primer sequences for RT-PCR.

Gene	Primer Sequences
β-actin	5’-AGCCTCGCCTTTGCCGA-3’
	5’-CTGGTGCCTGGGGCG-3’
EGR1	5’-GTTTGGCTGGGGTAACTGGT-3’
	5’-AGCCCTACGAGCACCTGAC-3’
HOXB9	5’-TCCAGCGTCTGGTATTTGGT-3’
	5’-GAAGCGAGGACAAAGAGAGG-3’
CRX	5’-GACACTGAGGCCCAAGTCAG-3’
	5’-TGTTTCCTTCAGCCTCTGCT-3’
E2F3	5’-CTAGCTCCAGCCTTCGCTTT-3’
	5’-AGCCTCCTCTACACCACGC-3’
GLIS2	5’-TGTGCTTAAAGCGGTCACTG-3’
	5’-AACCTGAAGATCCACAACCG-3’
STAT3	5’-CTGCTCCAGGTACCGTGTGT-3’
	5’-CCTCTGCCGGAGAAACAG-3’
TEAD1	5’-CTTGAATGTGCAATGAAGCG-3’
	5’-CGAAGTTTGCCTCGGACTC-3’
WT1	5’-TTGTGTGGTTATCGCTCTCG-3’
	5’-CAAATGACATCCCAGCTTGA-3’
ZNF217	5’-TCTCTTTTGTGCCATGCTGT-3’
	5’-TTGTGTGCCTGCTGGTAGTC-3’
ZNF263	5’-CTTTCCTCCTTGTCCCACCT-3’
	5’-CACGGTGCAGGAGAGTTATG-3’

Gene	Primer Sequences
IL6	5’-GTCAGGGGTGGTTATTGCAT-3’
	5’-AGTGAGGAACAAGCCAGAGC-3’
CXCL1	5’-CTTCCTCCTCCCTTCTGGTC-3’
	5’-GAAAGCTTGCCTCAATCCTG-3’
ITGB1	5’-CAGTCCAATCCAGAAAATTGG-3’
	5’-GAGTCGCGGAACAGCAG-3’
EFNA1	5’-TCAGCTGCACATGTATGGTGT-3’
	5’-TCTCTTGGGTCTGTGCTGC-3’
ITGAV	5’-TCCAAACCACTGATGGGACT-3’
	5’-GTGACTGGTCTTCTACCCGC-3’
NRP1	5’-TTGCAGTCTCTGTCCTCCAA-3’
	5’-GAAAAATGCGAATGGCTGAT-3’
FGF2	5’-AGCCAGGTAACGGTTAGCAC-3’
	5’-GGAGAAGAGCGACCCTCAC-3’
IL8	5’-AAATTTGGGGTGGAAAGGTT-3’
	5’-TCCTGATTTCTGCAGCTCTGT-3’
HIF1α	5’-TGGCTGCATCTCGAGACTTT-3’
	5’-GAAGACATCGCGGGGAC-3’
IL1β	5’-AAGCCCTTGCTGTAGTGGTG-3’
	5’-GAAGCTGATGGCCCTAAACA-3’
ITGA6	5’-GTTGGCTCTCTGCAGTGGAA-3’
	5’-CCTCTTCGGCTTCTCGCT-3’
CXCL2	5’-GCTTCCTCCTTCCTTCTGGT-3’
	5’-GGGCAGAAAGCTTGTCTCAA-3’
ITGAE	5’-CTATGCAGAGCAGAGTGTGGA-3’
	5’-TGAGGGGAAGCTGAGTGG-3’
VEGF	5’-AGCTGCGCTGATAGACATCC-3’
	5’-CTACCTCCACCATGCCAAGT-3’
FGF1	5’-AAGGTGGTGATTTCCCCTTC-3’
	5’-TGTGGAGAGAGGTACAGCCC-3’
TWIST1	5’-TCCATTTTCTCCTTCTCTGGAA-3’
	5’-GTCCGCGTCCCACTAGC-3’
ZEB2	5'-CAATACCGTCATCCTCAGCA-3'
	5'-CCAATCCCAGGAGGAAAAAC-3'
miR-192 promoter (methylated); HeyA8	5’-ATGGGATATTTTTTAGAGTTTTGTC-3’
	5’-CTTCTTCGTAAATACTAACCCTCG-3’

Gene	Primer Sequences
miR-192 promoter (unmethylated); HeyA8	5'-GGGATATTTTTTAGAGTTTTGTTGA-3'
	5'-TTCTTCATAAATACTAACCCTCACC-3'
miR-192 promoter (methylated); SKOV3ip1	5'-GTTTCGGTTTTTAATTGGTTTTC-3'
	5'-CTTCTTCGTAAATACTAACCCTCG-3'
miR-192 promoter (unmethylated); SKOV3ip1	5'-GTTTTGGTTTTTAATTGGTTTTTGT-3'
	5'-CTTCTTCATAAATACTAACCCTCACC-3'
ChIP - miR-192	5'-TCACCGAGGCTTCTTATCATC
	5'-GACTTTGCCCAGGAAGGAG
ChIP - IL6	5'-GCGATGGAGTCAGAGGAAAC-3'
	5'-GGCTAGAATTTAGCGTTCCAG-3'
ChIP - EFNA1	5'-AGACCCACAAGTGAGCGAAC-3'
	5'-TGCAGGAAAGAGCGAAGC-3'
ChIP - IL1β	5'-TTTCTCAGCCTCCTACTTCTGC-3'
	5'-TTGTGCCTCGAAGAGGTTTG-3'
ChIP - ITGA6	5'-CCCACATCTGCAAGAACAGG-3'
	5'-AGCGAGGGAGACTTTACTACCC-3'
ChIP - Control	5'-TTGCCAGTGGTGCATACAG-3'
	5'-CACTTCGGGAAGGTTGTTG-3'
